# An Integrated World Modeling Theory (IWMT) of Consciousness: Combining Integrated Information and Global Neuronal Workspace Theories With the Free Energy Principle and Active Inference Framework; Toward Solving the Hard Problem and Characterizing Agentic Causation

**DOI:** 10.3389/frai.2020.00030

**Published:** 2020-06-09

**Authors:** Adam Safron

**Affiliations:** Indiana University, Bloomington, IN, United States

**Keywords:** consciousness, free energy principle, active inference, generative model, autonomy, integrated information theory, global workspace, autoencoder

## Abstract

The Free Energy Principle and Active Inference Framework (FEP-AI) begins with the understanding that persisting systems must regulate environmental exchanges and prevent entropic accumulation. In FEP-AI, minds and brains are predictive controllers for autonomous systems, where action-driven perception is realized as probabilistic inference. Integrated Information Theory (IIT) begins with considering the preconditions for a system to intrinsically exist, as well as axioms regarding the nature of consciousness. IIT has produced controversy because of its surprising entailments: quasi-panpsychism; subjectivity without referents or dynamics; and the possibility of fully-intelligent-yet-unconscious brain simulations. Here, I describe how these controversies might be resolved by integrating IIT with FEP-AI, where integrated information only entails consciousness for systems with perspectival reference frames capable of generating models with spatial, temporal, and causal coherence for self and world. Without that connection with external reality, systems could have arbitrarily high amounts of integrated information, but nonetheless would not entail subjective experience. I further describe how an integration of these frameworks may contribute to their evolution as unified systems theories and models of emergent causation. Then, inspired by both Global Neuronal Workspace Theory (GNWT) and the Harmonic Brain Modes framework, I describe how streams of consciousness may emerge as an evolving generation of sensorimotor predictions, with the precise composition of experiences depending on the integration abilities of synchronous complexes as self-organizing harmonic modes (SOHMs). These integrating dynamics may be particularly likely to occur via richly connected subnetworks affording body-centric sources of phenomenal binding and executive control. Along these connectivity backbones, SOHMs are proposed to implement turbo coding via loopy message-passing over predictive (autoencoding) networks, thus generating maximum a posteriori estimates as coherent vectors governing neural evolution, with alpha frequencies generating basic awareness, and cross-frequency phase-coupling within theta frequencies for access consciousness and volitional control. These dynamic cores of integrated information also function as global workspaces, centered on posterior cortices, but capable of being entrained with frontal cortices and interoceptive hierarchies, thus affording agentic causation. Integrated World Modeling Theory (IWMT) represents a synthetic approach to understanding minds that reveals compatibility between leading theories of consciousness, thus enabling inferential synergy.

## Introduction and Background

Here, I introduce *Integrated World Modeling Theory (IWMT)* as a synthetic approach to understanding consciousness, using the *Free Energy Principle and Active Inference Framework (FEP-AI)* (Friston et al., 2006, 2017a; Friston, 2010) to combine multiple theories into a unified perspective. IWMT focuses on *Integrated Information Theory (IIT)* (Tononi, 2004; Tononi et al., 2016) and *Global Neuronal Workspace Theory (GNWT)* (Baars, 1993; Dehaene, 2014) as two of the most well-known theories of consciousness. Areas of agreement and disagreement between IIT and GNWT will be explored, as well as the extent to which points of contention might be productively addressed by situating these theories within FEP-AI. I then review the fundamentals of FEP-AI as a general systems theory, including points of intersection with IIT as an account of causal emergence. I then go on to discuss mechanistic and computational principles by which these theories can all be integrated using IWMT. In brief, IWMT states that consciousness may be what it is like to be processes capable of generating integrated models of systems and worlds with spatial, temporal, and causal coherence. IWMT further suggests that such coherence is only likely to be attainable for embodied agentic systems with controllers capable of supporting complexes of high degrees of integrated information, functioning as global workspaces and arenas for Bayesian model selection. Finally, I consider potential implications of these proposals with respect to the enduring problems of consciousness and artificial intelligence.

### Toward Integration

How can physical systems generate subjective experiences? Can mental states function as causes, or are we mere automata? These perennial questions may finally be answerable with two unifying frameworks for understanding complex systems and minds: FEP-AI and IIT. These two meta-theoretical frameworks were developed in the context of understanding psychological and neurobiological phenomena, yet their implications are far more extensive. FEP-AI may be the first unified formalism and paradigm for the mind and life sciences, and IIT is one of the most widely known and technically detailed models of consciousness and informational synergy. FEP-AI describes what systems must be like in order to persist, and IIT describes what it means for systems to intrinsically exist as systems. Both FEP-AI and IIT constitute general systems theories with scopes transcending disciplinary boundaries, having relevance not only for the philosophy and science of mind but also for understanding all emergent complexity.

Here, I describe how these two frameworks complement each other as unified systems theories, and also show how FEP-AI allows IIT and GNWT to be combined into a synthetic framework for understanding consciousness: IWMT. This synthesis further attempts to characterize the nature of mental causation in terms of generalized Darwinism (Campbell, 2016) and thermodynamic work cycles, thus describing how conscious agency may be essential for understanding how flexible intelligence may be realized in biological (and potentially artificial) systems. Toward this end, I attempt to address consciousness and autonomy on functional, algorithmic, and implementational levels of analysis (Marr, 1983). Finally, I discuss implications of theories of consciousness for the enduring problems of artificial intelligence.

### The Enduring Problems of Consciousness

How could there be “*something that it is like*” to be a physical system (Nagel, 1974; Lycan, 1996)? In introducing the *Hard problem*, Chalmers (1997) contrasted this question with the “easy problem” of understanding how biological processes contribute to different psychological phenomena. Proponents of the Hard problem argue that we could have a complete cognitive science, and yet still not understand consciousness. Could cognition take place “in the dark” without generating any subjective experiences, or *qualia*? Could such philosophical zombies perform all the computations enabled by brains, yet lack subjectivity?

Intellectual positions on these matters range from the more inflationary claim that consciousness is a fundamental aspect of the universe, to the more deflationary claim that the Hard problem will be (dis-)solved by answering the easy problems of cognitive science (Dennett, 2018), with no “explanatory gap” remaining. Others have suggested that these metaphysical questions distract from the more productive endeavor of studying why particular experiences are associated with particular physical processes: i.e., the “real problem” of consciousness (Seth, 2016). Even disagreement about the generation of the Hard problem has become a topic of philosophical inquiry and has been named the “*meta-problem*” (Chalmers, 2018).

While numerous models have been suggested, none are generally considered to have solved the enduring problems of consciousness. Such a solution would require explanation spanning implementational, algorithmic, and functional levels of analysis, with rich connections to experience. Here, I suggest that this multi-level understanding can be obtained by using FEP-AI to ground and combine leading models of consciousness into a unified framework centered on integrated world modeling (IWMT). This article focuses on IIT and GNWT, and in forthcoming work, I will extend this synthesis to additional models—e.g., Higher-Order Thought theories (Brown et al., 2019; Graziano, 2019; Shea and Frith, 2019)—each of which emphasizes different aspects of the nature(s) of consciousness.

Yet another enduring problem can be found in that there is no clearly agreed upon definition of consciousness. Some theories focus on consciousness as phenomenal experience. Others emphasize consciousness as awareness of knowledge, or “access” (Block, 2008). IWMT's primary focus is explaining means by which biological systems may generate phenomenality, or experience as a subjective point of view (Williford et al., 2018; Feiten, 2020). However, IWMT suggests that a variety of higher-order and meta-cognitive capacities may be required in order to obtain coherent subjectivity—although not necessarily involving either access or explicit self-consciousness (Milliere and Metzinger, 2020)—and thereby an experienced world. More specifically, IWMT's primary claims are as follows:

1. Basic phenomenal consciousness is what it is like to be the functioning of a probabilistic generative model for the sensorium of an embodied–embedded agent.
2. Higher order and access consciousness are made possible when this information can be integrated into a world model with spatial, temporal, and causal coherence. Here, coherence is broadly understood as sufficient consistency to enable functional closure and semiotics/sense-making (Joslyn, 2000; Pattee, 2001; Ziporyn, 2004; Gazzaniga, 2018; Chang et al., 2019). That is, for there to be the experience of a world, the things that constitute that world must be able to be situated and contrasted with other things in some kind of space, with relative changes constituting time, and with regularities of change constituting cause. These may also be preconditions for basic phenomenality (#1), especially if consciousness (as subjectivity) requires an experiencing subject with a point of view on the world.
3. Conscious access—and possibly phenomenal consciousness—likely requires generative processes capable of counterfactual modeling (Friston, 2018; Pearl and Mackenzie, 2018; Kanai et al., 2019; Corcoran et al., 2020) with respect to selfhood and self-generated actions.

In what follows, I attempt to justify these claims by integrating across leading theories of emergent causation and consciousness. This approach draws on the explanatory breadth and embodied cybernetic grounding of the FEP-AI, the focus on irreducible integrative complexity provided by IIT, and the functional and mechanistic details provided by GNWT. IWMT tries to make inroads into the enduring problems of consciousness by synergistically combining the relative strengths (and diverse perspectives) of these theories (Table 1).

**Table 1:** Comparisons between four perspectives on aspects of consciousness: FEP-AI, IIT, GNWT, and IWMT.

	**FEP-AI**	**IIT**	**GNWT**	**IWMT**
**Levels of analysis emphasized**	Functional, algorithmic, and implementational	Phenomenological and implementational	Functional and implementational	Phenomenological, functional, algorithmic, and implementational
**Emphasizes either phenomenal or access consciousness**	Both	Phenomenal	Access	Both
**Emphasizes either intrinsic or extrinsic perspectives**	Both	Intrinsic	Extrinsic	Both
**Neural substrates of consciousness**	A distributed pattern of effective connectivity (entailing Bayesian beliefs) across a multi-level deep temporal hierarchy, primarily generated by L5 pyramidal neurons and thalamic relays	A maximal nexus of self-cause–effect power, likely centered on posterior cortices	A global workspace realized by re-entrant connectivity between frontal and posterior cortices	Agreement with FEP-AI, except these distributed patterns are hypothesized to be integrated via the formation of self-organizing harmonic modes, so promoting communication through coherence Agreement with IIT with respect to basic phenomenal consciousness, but with specific emphasis on posterior-medial cortices as a basis for egocentric perspective Agreement with GNWT with respect to access consciousness, but with phenomenality being generated from posterior loci
**Minimally conscious system**	Any generative model with temporal depth and counterfactual richness; e.g., all deep belief hierarchies capable of adaptive active inference	Any system capable of generating irreducible cause–effect power over itself; e.g., a single elementary particle	Any system capable of implementing a global workspace; e.g., a computer program with a blackboard architecture	Any process capable of generating a world model with spatial, temporal, and causal coherence with respect to the system and its causal inter-relations with its environment; e.g., all mammals, possibly all vertebrates, and possibly insects
**Can a system without dynamics be conscious?**	No	Yes, if it is part of a configuration capable of constraining likely past and future states	No	No
**Could an artificial intelligence (AI) implemented on a von Neumann architecture be conscious?**	Yes	No	Yes	Probably
**Is either physical or a richly structured virtual embodiment required for consciousness?**	Yes	No	No	Yes
**Associated concepts from machine learning and AI**	Variational autoencoders Forney factor graphs with marginal message passing	Direct implementation on neuromorphic hardware capable of recurrent dynamics	Blackboard architectures	Folded variational autoencoders with recurrent dynamics Turbo codes
**Are human-equivalent intelligent zombies feasible?**	No comment	Yes	No comment	Theoretically conceivable, but practically infeasible

*FEP-AI, Free Energy Principle and Active Inference framework; IIT, Integrated Information Theory; GNWT, Global Neuronal Workspace Theory; IWMT, Integrated World Modeling Theory*.

### IWMT: Combining IIT and GNWT With the FEP-AI

This section provides an introduction to FEP-AI, IIT, and GNWT, as well as an initial account of how they may be combined within IWMT. Further details regarding FEP-AI and IIT are explored in subsequent sections, followed by a further integration with GNWT.

#### FEP-AI

The Free Energy Principle states that persisting systems must entail predictive models to resist entropic mixing (Friston, 2019). That is, to prevent destruction and maintain their forms, systems must adaptively respond to a variety of events, and so must be able to model these events in some capacity (Conant and Ashby, 1970). Beginning from this fundamental principle of nature (Hohwy, 2020), the FEP and Active Inference (FEP-AI) framework (Friston et al., 2017a) proscribes means of satisfying this imperative through minimizing prediction-error (or “free energy”) with respect to the models by which systems preserve themselves. In contrast to views in which experience emerges from passive sensations, FEP-AI understands perception as taking place within the context of actions, including foraging for information and resolving model uncertainty. Within this framework, both perception and action are understood as kinds of predictions/inferences regarding the means by which prediction-error might be minimized (hence, “active inference”).

*Hierarchical predictive processing (HPP)* offers powerfully explanatory implementational and algorithmic details for active inference (Clark, 2016), providing a single mechanism for both perception and action. FEP-AI further emphasizes the roles of embodiment, selfhood, and agency in minimizing free energy via action–perception cycles, thus naturally supporting bridges to phenomenology on multiple levels. While probabilistic modeling may narrow explanatory gaps between brain and mind, the question remains: how do (seemingly definite) subjective experiences emerge from probabilities?

#### IIT: Informational Synergy Through Balanced Integration and Differentiation; of MICE and MAPs

IIT begins from phenomenology (Tononi, 2004), observing that consciousness is distinct in its particular details (i.e., information), while also being experienced holistically (i.e., integration). This observation generated the hypothesis that consciousness results from the ability of nervous systems to support diverse state repertoires, while also synergistically integrating this information into wholes greater than the sum of their parts. IIT further suggests that this capacity for generating integrated information can be quantified by analyzing the degree to which systems are irreducible to the information contained in their parts considered separately. IIT has developed through multiple iterations, most recently formalized with phenomenological axioms and the postulated properties required for realizing these aspects of experience in physical systems (Tononi et al., 2016). These postulates are stipulated to be not only *necessary*, but also, controversially (Bayne, 2018; Lau and Michel, 2019), *jointly sufficient* conditions for conscious experience (Table 2).

**Table 2:** Integrated Information Theory (IIT) axioms and postulates, with corresponding examples of experiences and mechanistic systems.

**IIT axioms: Essential properties of experience**	**Example experiences**	**IIT postulates: Properties of physical systems capable of accounting for experience**	**Example systems**
**Intrinsic existence:**			
Experience exists from its own intrinsic perspective (i.e., subjectivity), independent of external observers.	My experience of a red apple has intrinsic existence in that it is both real to me and also private.	A system has cause–effect power upon itself; present states must inform probabilities of past and future states, so linking causes and effects.	A brain has internal connectivity that influences which states are likely to flow from the past to the future, given its present state; some parts of brains have more intrinsic connectivity than others.
**Composition:**			
Experience is structured by the elementary or higher-order subjective distinctions out of which it is composed.	My experience of a red apple is composed of particular features, such as redness for color and apple shape for form.	A system is structured by the more elementary sub-systems out of which it is composed, and which have cause–effect power upon the system.	A brain is composed of neurons, whose particular configurations influence its past and future states; different parts of brains have different compositions.
**Information:**			
Experience is particular in being composed of a specific set of subjective distinctions, so being differentiated from other possible experiences.	My experience of a red apple is informative in being perceived in terms of particular qualities of subjective redness (as opposed to greenness) and apple shape (as opposed to pear shape).	A system specifies a particular cause–effect structure that informs particular probabilistic repertoires of past causes and future effects for the system and sub-systems, so differentiating particular states from other possible states.	A brain can be configured in many different ways, and so any particular configuration is highly informative in terms of being distinguished from other possible configurations; some parts of brains are more informative than others in different contexts.
**Integration:**			
Each experience is unified in being irreducible to independent subsets of subjective distinctions.	My experience of a red apple is integrated in that redness and apple shape are not independently perceived, but are instead combined into a unified whole.	A system specifies a unified cause–effect structure that is irreducible to independent sub-systems (phi > 0), including its minimally interdependent component.	A brain has properties that do not exist in its individual neurons considered separately; some parts of brains are more integrated than others in different contexts.
**Exclusion:**			
Each experience is definite in content and spatiotemporal grain, specifying a particular set of subjective distinctions unfolding on particular spatiotemporal scales.	My experience of a red apple has particular contents with respect to space and time, with particular redness and apple shape being perceived at some spatiotemporal scales and not others.	A system specifies particular cause-effect repertoires over particular sets of elements at particular spatial and temporal grains. The boundaries of a system are defined by a complex entailing a maximally irreducible conceptual structure (MICS) existing at particular spatial and temporal grains, whose total integrated information is quantified as Phi-max.	A brain and its sub-systems have particular boundaries that determine the extent to which they function as integrative wholes in space and time; some parts of brains have clearer boundaries than others in different contexts (e.g., modularity).

IIT is both a theory of consciousness and meta-physical formalism, attempting to answer the question: what counts as a system from an intrinsic perspective (Fallon, 2018)? IIT models systems as networks of causal relations, evaluating compositional structures for their ability to inform (or constrain) past and future states. *Integrated information (phi)* is calculated based on the degree to which cutting systems along a *minimum information partition (MIP)* impact past and future self-information, evaluated across all relevant spatial and temporal grains for system evolution. The extent to which MIPs reduce self-information is used to calculate the degree to which systems make irreducible (i.e., integrated) causal differences to themselves, thus defining their integrated information (quantified as phi). Intuitively, if something can be decomposed into parts without consequence, then it is not an integrated system. According to the exclusion axiom, systems are only real (and potentially conscious) if they represent maxima of integrated information. The self-directed causal relation of a maximal complex is referred to as a *maximally irreducible conceptual structure (MICS)*—corresponding to mappings onto an abstract metric space (i.e., “qualia space”) (Balduzzi and Tononi, 2009), whose particular geometries correspond to particular experiences. Further, sub-mechanisms contributing given MICS will be associated with a variety of phenomenal distinctions, specified as *maximally irreducible cause-effect (MICE) repertories*.

While IIT's experience-first approach provides compelling bridges between phenomenology and mechanistic implementations, the question remains: why should there be “anything that it is like” to be a maximally irreducible cause-effect structure? As described below, IWMT proposes that a maximal complex (entailing a MICS) could also entail subjective experience, *if (and only if)* these complexes also entail probabilistic mappings—or maximal a posteriori (MAP) estimates derived thereof—entailed by generative models for the sensoriums of embodied–embedded goal-seeking agents. As described in further detail below, IWMT further proposes that phi parameterizes the ability of systems to minimize free energy and maximize self-model evidence. While the most valid means of defining integrated information for conscious (or unconscious) systems remains contested (Barrett and Mediano, 2019), one potential advance from IWMT's proposed synthesis could be identifying the appropriate uses for various formulations of integrative complexity.

The putative sufficiency of IIT's phenomenological postulates for consciousness results in a surprising implication: the degree to which systems exist is also the degree to which they generate conscious experience (Tononi and Koch, 2015). As will be described in greater detail below, IWMT accepts a modified version of this proposition with fewer protopansychist implications: systems exist to the degree they generate model evidence for themselves, which may entail consciousness if models have spatial, temporal, and causal coherence for systems and world. Below, I describe how systems might be configured if they are to generate complexes of integrated information with these coherence-enabling properties.

*[Note: A more detailed discussion of IIT's postulates and axioms can be found in IWMT Revisited (Safron*, *2019a**), in the section: “A review of IIT terminology.”]*

#### GNWT: Functional Synergy Through Balancing Integrated and Segregated Processing; Critical Modes of Consciousness as Bayesian Model Selection

Originally introduced by Baars (1993), Global Workspace Theory considers computational requirements for intelligent functioning, drawing analogies between consciousness and computing architectures in which “blackboards” share information among multiple specialist processes. According to Baars, consciousness is hypothesized to correspond to a “global workspace” that allows unconscious segregated processes to communicate with informational synergy. Information becomes conscious when it enters workspaces, and so can be effectively broadcast throughout entire systems. Because of workspaces' limited capacities, specialist processes compete and cooperate for selection based on abilities to satisfy context-specific computational objectives. Workspace architectures have been used in artificial intelligence (Hofstadter and Mitchell, 1994; Shanahan and Baars, 2005; Madl et al., 2011) because of their capacity for integrative functioning with competition-enhanced efficiency. These systems have also been configured in ways that recapitulate notable psychological phenomena, including cognitive cycles involving separable phases of sensing, interpreting, and acting.

The ability of workspaces to “select” value-enhancing information was interpreted as instantiating a quasi-Darwinian process by Edelman et al. (2011). According to neural Darwinism, the functionality of global workspaces provides a computational-level description of a mechanistic “dynamic core,” which promotes activity for particular neuronal ensembles through re-entrant connectivity. In line with theories emphasizing binding via synchronous dynamics (Singer, 2001; Varela et al., 2001; Crick and Koch, 2003), the thalamocortical system has been suggested to play key roles in this value-dependent selection and broadcasting of neuronal information.

In terms of neuronal architecture, van den Heuvel and Sporns (2011) have identified connectomic “rich club” networks, whose high centrality and interconnectivity may allow systems with mostly local connections to achieve both integrated and differentiated processing (Sporns, 2013). Shanahan (2012) has further noted that these core networks may be related to intelligence—and presumably consciousness—in non-human animals. Intriguingly, with respect to global workspaces, varying degrees of functional connectivity between richly connected networks have been found to be accompanied by periods of either high or low modularity (Betzel et al., 2016), consistent with a potential functional significance of integrating information across otherwise isolated sub-systems. More recent work (Esfahlani et al., 2020) has demonstrated that transient periods of strong co-activation within these networks explains much of the overall variance and modularity with respect to network structures, consistent with alternating periods of integration and segregation via workspace dynamics.

Within this paradigm of consciousness as enabling the integration and broadcasting of information, Dehaene (2014) has made invaluable contributions in describing how biological implementations of workspace dynamics may help to explain otherwise mysterious aspects of cognition (e.g., psychological refractory periods, attentional blinks). Dehaene et al. have also characterized time courses for unconscious and conscious information processing, showing how transitions to conscious awareness correspond to non-linear increases in large-scale brain activity. These “ignition” events are stipulated to indicate the accumulation of a critical mass of mutually consistent information—implemented by converging excitatory neural activity—so selecting one interpretation out of multiple possibilities. This neurobiological account in which neuronal systems dynamically move between more integrated and segregated processing is referred to by Dehaene and Changeux (2005) as GNWT. From an FEP-AI (and IWMT) perspective, these phase transitions may correspond to discrete updating and Bayesian model selection with respect to perception and action (Friston et al., 2012a; Hohwy, 2012; Parr and Friston, 2018b). GNWT has been increasingly described in terms of Bayesian inference (Dehaene, 2020; Mashour et al., 2020), including in a recently proposed Predictive Global Neuronal Workspace model (Whyte and Smith, 2020).

If neural dynamics can select particular interpretations of events, formally understood as Bayesian inference, then we seem even closer to closing explanatory gaps between mind and brain. Yet, the enduring problems of consciousness remain: Why should it be (or “feel”) like something to be a probabilistic model, and which biophysical processes specifically enable workspace-like dynamics?

#### FEP-AI + IIT + GNWT = IWMT

IIT focuses on consciousness as emerging from systems that are both unified and differentiated through their internal cause–effect relations. GNWT focuses on consciousness as emerging from systems that allow both global and local processing to be balanced through cycles of selecting, amplifying, and broadcasting information. In these ways, IIT and GNWT have identified highly similar preconditions for subjective experience.

While there are extensive similarities between GNWT and IIT, there are also notable differences (Table 1). GNWT focuses on systems engaging in cognitive cycles of acting and perceiving. This focus on integrative agentic functioning is highly compatible with the enactive bases of FEP-AI, where action–perception cycles are driven by rounds of Bayesian model selection. IIT has a broader scope, ascribing consciousness to all systems self-governed by emergent causes. As discussed below, this suggestion is partially compatible with FEP-AI, albeit with a restricted interpretation of the meanings of integrated information as potentially being necessary, but not sufficient for consciousness (Lau and Michel, 2019).

With respect to the neural substrates of consciousness, IIT identifies a “posterior hot zone” (Boly et al., 2017), which has been stipulated to represent a maximum of phi in the brain (Boly et al., 2017), and potentially also a source of spatial phenomenology, due to its organization as a hierarchy of 2D grids (Haun and Tononi, 2019). [Note: This stipulation is currently purely theoretical, as the computations required to formally identify maximal complexes are intractable for biological systems, and it remains contested which estimation methods are most valid in which contexts (Mediano et al., 2019b).] GNWT, in contrast, suggests that consciousness and global availability are made possible by connectivity between posterior and frontal regions. IWMT considers both positions to be accurate, but with respect to basic phenomenal and access consciousness, respectively.

Some of this dispute regarding the neural substrates of consciousness could potentially be resolved by identifying multiple types of workspace (and integrating) dynamics. One way of achieving widespread availability may be via synchronous stabilization (Humphrey, 2017) of representations, or as I suggest below, via *self-organizing harmonic modes (SOHMs)*. These processes may center on posterior hot zones, with information taking the form of a distributed causal nexus with both intrinsic integrated information and extrinsic functional significance. Alternatively, availability may also be achieved via the re-representation and accessing of information. These processes may also center on posterior (particularly medial) cortices as substrates for abstract (low-dimensional) features, potentially providing the kinds of representations adduced by symbolic cognitive science. Global availability and meta-awareness for this information would depend on coupling with the frontal lobes—which would also provide goal-oriented shaping of dynamics—although phi maxima and experience itself might still be generated in posterior hot zones as loci for embodied simulation (Barsalou, 2008, 2009, 2010; Prinz, 2017).

*[Note: More details regarding neural substrates of consciousness are described below, as well as in IWMT Revisited* (Safron, 2019a) *in the sections: “Neural systems for coherent world modeling” and “Future directions for IIT and GWT.”]*

#### Selfhood, Autonomy, and Consciousness

By grounding IIT and GNWT within the body-centered perspective of FEP-AI, IWMT suggests that complexes of integrated information and global workspaces can entail conscious experiences *if (and only if)* they are capable of generating integrative world models with spatial, temporal, and causal coherence. These ways of categorizing experience are increasingly recognized as constituting essential “core knowledge” at the foundation of cognitive development (Spelke and Kinzler, 2007). In addition to space, time, and cause, IWMT adds embodied autonomous selfhood as a precondition for integrated world modeling. As suggested by Kant (1781) (cf. transcendental unity of apperception), Helmholtz (1878), Friston (2017), and others—e.g., von Uexküll (1957), Damasio (2012), and Humphrey (2017)—IWMT argues that integrated selfhood and autonomy are required for coherent sense-making. For there to be “something that it is like”—and even more so, “something it feels like”—workspace dynamics must be grounded in models of autonomous embodiment (Safron, 2019a,c).

With respect to autonomy, IWMT further suggests that driving of cognitive cycles by “ignition” events may be an apt description. That is, if workspace dynamics implement Bayesian model selection—driven by the minimization of free energy—then cognitive cycles may be fully isomorphic with both thermodynamic work cycles (Kauffman and Clayton, 2006; Deacon, 2011) and selective pressures in the context of generalized Darwinism (Kaila and Annila, 2008; Campbell, 2016; Safron, 2019b). That is, if ignition corresponds to large-scale updating and communication of Bayesian beliefs, then formally speaking, these events may be sources of cause–effect power in precisely the same ways that controlled explosions drive engines to generate work. If these beliefs entail intentions for acting and the phenomenology of willing, then will power may be a systemic cause and source of force in every meaningful sense of the words “power,” “cause,” and “force” (Carroll, 2016; Sengupta et al., 2016; Pearl and Mackenzie, 2018; Safron, 2019c; Friston et al., 2020b).

As described below, this connection to autonomy is yet another way in which IIT and GNWT may be synergistically combined: the ability of workspaces to support cognitive cycles may depend on maintaining coherent internal dynamics, which may also depend on exerting cause–effect power over themselves. With respect to IIT, maximally irreducible cause-effect structures (MICS) may correspond to maximally probable inferences over sensorimotor states for integrated systems, as well as sources of maximal control energy governing system evolution. Thus, IWMT's cybernetic (Seth, 2015; Safron, 2019c) grounding of IIT and GNWT within FEP-AI may not only help explain why there may be “something that it is like” to be a maximal complex (entailing a MICS and MICE repertoires), but also provide causal connections between consciousness and action, thus providing foundations for the emergence of agency (Tononi, 2013).

The *default mode network (DMN)* and functional networks with which it interacts (Huang et al., 2020) may be particularly important for understanding the emergence of both phenomenal and higher-order consciousness, and also agency. In predictive processing, intentional action selection requires an ability to maintain counterfactual predictions in the face of otherwise inconsistent sense data (Safron, 2019c). However, driving systems into otherwise uncharted territories of inference-space will involve temporary local increases in prediction-error (i.e., “free energy”) for portions of generative models that recognize discrepancies between imagined goal states and current sensory observations. In order for goal-oriented behavior to proceed, this free energy must be buffered by other systems capable of acting as temporary thermodynamic reservoirs (Carhart-Harris and Friston, 2010). The DMN and its imaginative capacities (Beaty et al., 2014, 2015, 2018; Hassabis et al., 2014) may instantiate this kind of (informational) creative dynamo, constituting sources of strongly internally coherent predictions, thus being capable of temporarily absorbing and then releasing free energy via the shaping of perception and driving of action. The network properties of the DMN are ideally suited to serve these functions, having both high centrality—and so high potential for integrating information and exerting control (Kenett et al., 2018)—while also being located distally from primary modalities, and so being capable of supporting dynamics that are more decoupled from immediate sensorimotor engagements (Sormaz et al., 2018; Corcoran et al., 2020). Further, the DMN is likely to support some of the most stable inferences available to embodied–embedded persons, with major nodes allowing for egocentric perspective—i.e., providing a subjective point of view in generating world models with spatial, temporal, and causal coherence—integrated memory, and even the foundations of selfhood (Dennett, 1992; Hassabis and Maguire, 2009; Northoff, 2012; Brewer et al., 2013; Davey and Harrison, 2018). Indeed, the DMN and the networks with which it couples may be well-modeled as a complex of effective connectivity with high degrees of integrated information, functioning as a dynamic core and global workspace for conscious imaginings (Wens et al., 2019). In these ways, and as will be described in greater detail below, IWMT suggests that a multi-level account of the nature of embodied experience and its connections to phenomenology may contribute to the quest for obtaining satisfying solutions to the Hard problem.

## FEP-AI and IIT: Unified Systems Theories

The following sections discuss FEP-AI and why it is increasingly recognized as a unified systems theory. I will also suggest ways that IIT can be integrated with FEP-AI, thereby illuminating the nature of consciousness and causal emergence more generally. Readers specifically interested in the neurocomputational bases of consciousness may want to skip to “Mechanisms of Integrated World Modeling.” However, this is not recommended, as earlier sections help to show how FEP-AI provides a multi-level grounding for other theories in fundamental biophysics, thus linking mind and life. These sections also help to clarify what is and is not implied by these frameworks (i.e., which systems are likely to have or lack consciousness), as well as the implications of their integration for understanding emergent complexity in multiple domains.

### Resisting the 2nd Law With Generative Modeling (and Integrated Information)

According to the 2nd law, systems should exhibit increasing disorder until they cease to exist. Yet some things do manage to (temporarily) persist, and so something about their configurations must organize environmental exchanges to avoid entropic accumulation (Schrodinger, 1944; Brillouin, 1951; Deacon, 2011; Ramstead et al., 2018). Persisting systems somehow generate dynamics that steer away from the maximally probable outcome of maximal disorder. In cybernetics and control theory, the requirements for such governing processes are expressed as the *good regulator theorem* and *law of requisite variety*: any effective controller must be able to (at least implicitly) model that system, and regulating models require sufficient complexity to represent the variety of states likely to be encountered (Conant and Ashby, 1970).

FEP-AI (Friston, 2019) views persisting systems as entailing generative models for the preconditions by which they persist. For a system to constitute a model, its composition must be able to either compress or predict information for that which is modeled. Persisting systems specifically generate mutual (probabilistic) information between past and future states based on their present compositions. These mappings between particular configurations and ensuing dynamics constitute likelihoods (as particular action tendencies), thus characterizing system compositions as generative models, which generate dynamics that maximize the probability of those particular compositions. If it were not the case that system configurations generate dynamics that maintain those configurations, then no persisting systems would exist. Thus, persisting systems can be viewed as generative models that generate evidence for themselves through their dynamics, and so engage in “*self-evidencing*” (Hohwy, 2016).

In this way, FEP-AI provides a formalization and generalization of autopoietic self-making as described by Maturana and Varela (1980):

“*An autopoietic machine is a machine organized (defined as a unity) as a network of processes of production (transformation and destruction) of components which: (i) through their interactions and transformations continuously regenerate and realize the network of processes (relations) that produced them; and (ii) constitute it (the machine) as a concrete unity in space in which they (the components) exist by specifying the topological domain of its realization as such a network.”*

To the degree systems persist, they possess attracting sets that define them as particular phase space densities—whose action constitutes trajectories through state space—with varying probabilities of occurrence. In autopoiesis, attractor dynamics produce the very mechanisms out of which they are generated. FEP-AI views these autopoietic attractor configurations and ensuing trajectories as self-predicting generative models (Palacios et al., 2020), where that which is generated is the very probabilistic densities that define the existence of particular systems.

FEP-AI goes on to quantify self-model evidence according to an information-theoretic functional of variational (or approximate) free energy (Dayan et al., 1995). Derived from statistical physics, this singular objective function is optimized by minimizing discrepancies between probabilistic beliefs and observations (i.e., prediction-error, or “surprisal”), penalized by model complexity. To the extent systems persist, they constitute existence proofs (Friston, 2018) that they were able to bound surprise (i.e., high-entropy configurations) relative to predictive models by which they perpetuate themselves. Systems must respond adaptively to a variety of situations in order to avoid entropy-increasing events, and so must entail models with sufficient complexity to predict likely outcomes, thus minimizing discrepancies between expectations and observations. However, these models must not have so much complexity that they waste energy or over-fit observations and fail to generalize their predictions (also, more complex models are more energetically costly to implement). Variational free energy provides an objective function that optimally balances these requirements for accuracy and simplicity.

The extreme generality of FEP-AI requires emphasis. Not only do nervous systems entail predictive models, but so do entire populations of organisms and their extended phenotypes (Dawkins, 1999) as teleonomical (Deacon, 2011; Dennett, 2017) predictions with respect to evolutionary fitness (Friston, 2018; Ramstead et al., 2018). By this account, nervous systems are merely a (very) special case of generative modeling, where *all systems are models* in their very existence, but where *some systems also have sub-models* that function as cybernetic controllers (Stepp and Turvey, 2010; Seth, 2015; Seth and Tsakiris, 2018). In these ways, FEP-AI provides a formalism where persisting dynamical systems can be understood as self-generating models, grounded in first principles regarding the necessary preconditions for existence in a world governed by the 2nd law.

This view of systems as self-predicting generative models has clear correspondences with IIT, since self-evidencing depends on capacity for generating self-cause–effect power. I suggest we should further expect model-evidence for system preservation to be related to a system's ability to function as a unified whole, and so integrated information maximization ought to accompany free energy minimization. Notably, IIT-based models of metabolic cycles and gene-regulatory networks—core processes for homeostasis and autopoiesis—suggest that adaptive capacities of biotic systems may require high-phi configurations (Marshall et al., 2017; Abrego and Zaikin, 2019). Systems with lower phi may be qualitatively different from systems with higher phi (Albantakis, 2017; Albantakis and Tononi, 2019), being less capable of state-dependent adaptation—and thereby learning—which may drastically limit their intelligence and agency. These IIT-informed studies are fully consistent with FEP-AI, wherein all persisting systems minimize free energy, but only evolved systems minimize expected free energy via generative models where causes can be modeled with temporal depth and counterfactual richness (Kirchhoff et al., 2018).

### An Ontology of Markov Blankets: Estimating Boundaries (and Intelligence-Potential) for Processes/Things as Self-Predicting Models and Complexes of Integrated Information

This formalization of autopoietic systems can also be derived with graphical modeling concepts, providing further bridges between FEP-AI and IIT. Graphical models represent systems as structured relationships among component variables and their connections. If these connected variables are associated with probabilities—whether due to uncertain observations or inherent stochasticity—then that representation is a probabilistic graphical model (PGM) (Koller and Friedman, 2009). PGMs specify probability distributions over variables, thus entailing probabilistic models of that which is represented. This mapping from connected graphs to probabilities allows PGMs to synergistically combine information from multiple sources. Integration into joint probability distributions affords inference of both likely beliefs from observations (i.e., discriminative models) and likely observations from beliefs (i.e., generative models). With importance for subsequent discussions of consciousness, these graphs not only enable the generation of probabilistic world models (i.e., inference) and refinements of these models with observations (i.e., learning), but PGMs also afford discrete estimates of the most likely values for variable combinations, as in maximum a posteriori (MAP) estimation.

For any PGM component, the set of surrounding nodes is referred to as a *Markov blanket (MB)* (Pearl, 1988), which establishes conditional independence between internal and external variables. All paths connecting internal and external states are mediated by MBs; thus, conditioning upon this blanketing set integrates all mutual information across this partition (i.e., marginalization). System MBs define epistemic relationships with the external world in providing the only source of information that internal states ever receive (Hohwy, 2017). Everything beyond MB boundaries is not directly observable, and so latent values of external states must be inferred.

Described as PGMs, the functional boundaries of systems are MBs (Kirchhoff et al., 2018), mediating all that can ever be known about or done to the outside world. Some examples: single-celled organism MBs are largely co-extensive with cellular membranes; nervous system MBs are composed of sensor and effector neurons by which they receive information from sensors and drive change with actuators; niche-constructing organism MBs constitute the boundaries of extended phenotypes, including bodies and external structures that regulate environmental interaction. Such functional boundaries are an essential source of adaptive constraints for biological systems (Rudrauf et al., 2003; Hordijk and Steel, 2015; Lane, 2016), both internally concentrating system-promoting complexity and limiting system-threatening exchanges with external environments. Thus, MBs are both epistemic and system-defining boundaries. With respect to IIT, the boundaries of maximal complexes (entailing maximally irreducible cause-effect structures) would also constitute MBs. Although each MICS represents a kind of world unto itself (Leibniz, 1714), FEP-AI's formalism of internal states as modeling external states (and vice versa) may provide a means of understanding how such inwardly directed phenomena can nonetheless come to “encode” meaningful information about the external world with which they co-evolve, potentially providing linkages between IIT's intrinsic integrated information and information theory more generally.

The dual epistemic and ontological roles of MBs help justify the extremely broad scope of both FEP-AI (and possibly IIT as well). Identifiable systems must have boundaries defining their extents relative to other systems. Persisting systems further require predictive models to maintain themselves and their MB boundaries as they interact with environments. Yet, because blanket states informationally shield internal states from the rest of the world, modeling external states and MB boundaries necessitates inference (Friston, 2017, 2018, 2019). In this way, the epistemic boundaries created by system-defining MBs require persisting dynamical systems to entail self-evidencing generative models.

### Generative Modeling, Integrated Information, and Consciousness: Here, There, but Not Everywhere?

The extreme generality of PGMs and the implicit modeling relationships prescribed by FEP-AI may be of an extremely simple variety, particularly if systems have limited dynamic character and restricted thermodynamic openness. To provide an intuition-stretching example, by virtue of persisting (and so generating model evidence for their existence), the configuration of rocks and resultant causal interactions could be viewed as instantiating an implicit “prediction” that intramolecular forces and limited exchanges will be sufficient to maintain their forms. On short timescales, rocks will be able to (non-adaptively) generate rock-like dynamics, which restrict thermodynamic exchanges, thus allowing rocks to temporarily avoid disintegration. However, in contrast to living systems, rocks lack functional closure (Joslyn, 2000; Pattee, 2001; Deacon, 2011; Gazzaniga, 2018) with the geological processes generating their forms. Without multi-level evolutionary optimization (Safron, 2019b), generative models will be of such simple varieties that they are incapable of predicting and responding to particular events (i.e., adaptation). In this way, rocks are “surprised” by every exchange with their environments capable of altering their structures, and so will steadily disintegrate as such exchanges accumulate over time. [Note: FEP-AI focuses on weakly mixing ergodic systems, and as such, this conceptual analysis of rocks lacks the kinds of formal treatments that have been—controversially (Biehl et al., 2020; Friston et al., 2020a)—applied to complex adaptive systems.]

This consideration of rocks as (very) impoverished generative models provides a limit case for understanding what is and is not implied by FEP-AI: every ‘thing' can be viewed as having a basic kind of intelligence by virtue of existing at all, but neither rocks nor other similar inanimate objects are conscious (Friston, 2018, 2019). This limit case also shows major points of intersection between FEP-AI and IIT (Table 1), as both frameworks provide universal ontologies, and so must be applicable to every system, including rocks, and potentially even the processes giving rise to physical forces and their associated particles (Tegmark, 2014). However, according to IIT's exclusion axiom, rocks would not represent actual systems, in that maxima of integrated information would likely be found among separate components, and so neither (intrinsic) existence nor quasi-sentience would be ascribed. While the exclusion axiom may be essential for consciousness, relaxing this postulate in some cases may allow IIT to both (a) be fully compatible with FEP-AI and (b) better function as a general model of emergent causation. That is, for something to be said to exist, it may not be necessary for it to be a maximum of integrated information as irreducible cause–effect power. Rocks do indeed exist—while lacking consciousness—in that they possess emergent properties that are not present in their constituent elements (e.g., the intrinsic property of a boulder being able to maintain its form as it rolls (Bejan, 2016), or its extrinsic properties with respect to anything in the path of a large quickly moving object). Large-scale compositions may not represent maximal complexes, but may nonetheless play important roles with respect to internal functioning and interactions with other systems.

With respect to the exclusion principle, IIT theorists have suggested that advanced artificial intelligences could be unconscious “zombies” if deployed on von Neumann architectures (Tononi and Koch, 2015), which lack irreducible integration due to serial operation. However, alternative interpretations of IIT could extend phi analyses into temporally extended virtual processes, rather than solely focusing on “direct” realization by physical mechanisms. From an FEP-AI perspective, maximally explanatory models for computer programs may correspond to (MB-bounded) functional cycles on the software level. This proposal for updating IIT aligns with a recently-suggested theory of consciousness focusing on spatiotemporal scales at which functional closure is achieved (Chang et al., 2019), thus instantiating emergence and affording coarse-graining over lower levels of analysis. However, both Information Closure Theory and IIT purport that consciousness corresponds to any instance of emergent causation. IWMT, in contrast, argues that consciousness may be “what physics feels like from the inside” (Koch, 2012; Tegmark, 2014), *if (and only if)* physical processes support the generation of integrated system–world models with spatial, temporal, and causal coherence.

### Consciousness, Emergence, Integrative Synergy

**Figure 1:**
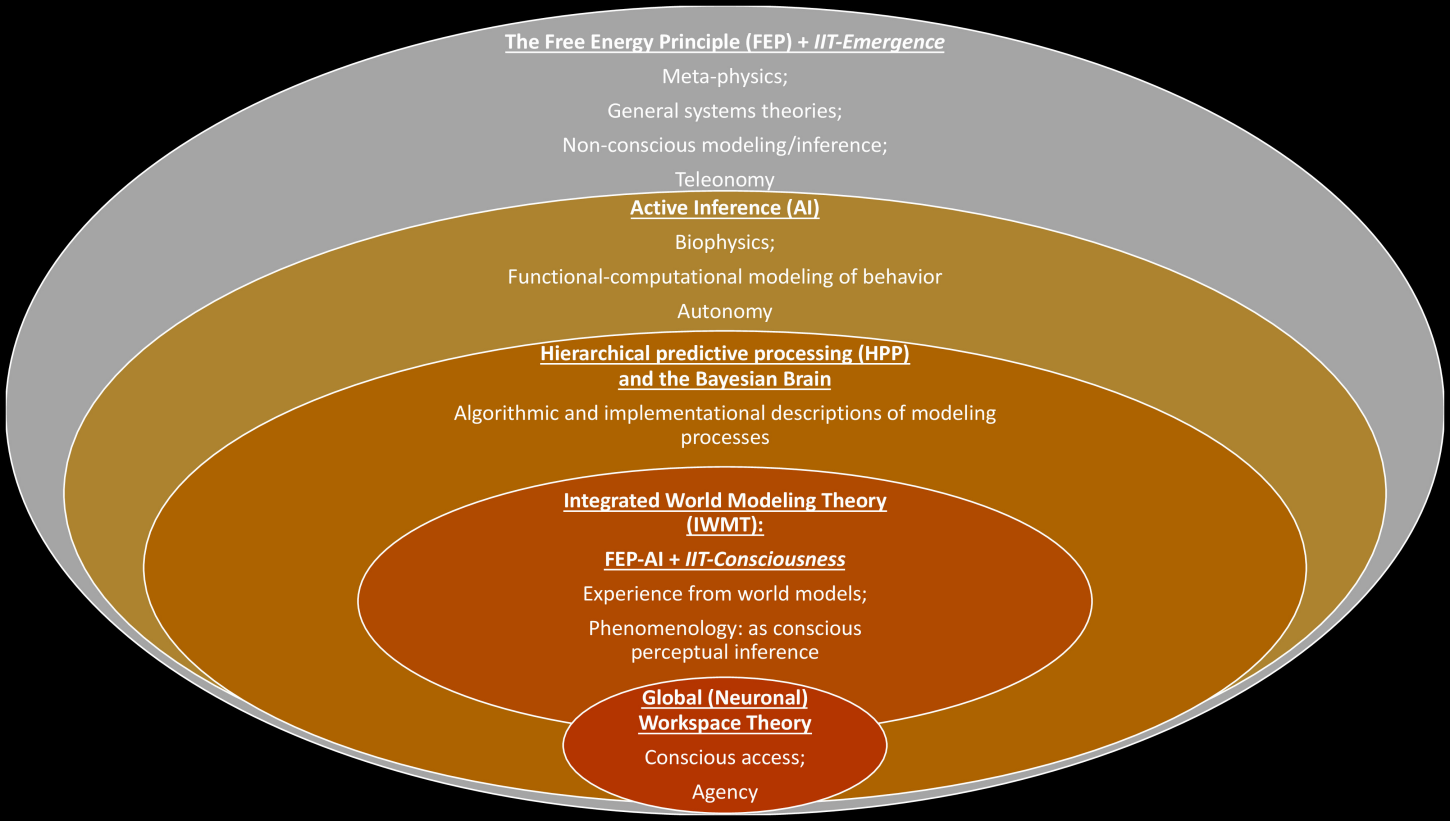
Intersections between FEP-AI, IIT, GNWT, and IWMT.

IWMT suggests that leading theories of consciousness can be synergistically combined within FEP-AI. FEP-AI and IIT both play dual roles in this synthesis, serving as both general systems theories and descriptions of the processes underlying subjective experience. FEP-AI and IIT intersect on multiple levels, with potential for understanding causal emergence on multiple scales. However, the nature of these explanations may vary across domains, including with respect to analytic assumptions. Integrated information may potentially be modeled in different (and differently valuable) ways in different contexts (Tegmark, 2016; Mediano et al., 2019a,b), which may range from the identification of natural kinds, to the nature of life, to perception, and even consciousness (Figure 1). Based on these considerations, I propose it may be productive to factorize IIT into two complementary versions:

1. IIT-Consciousness: the original version of the theory.
2. IIT-Emergence: an alternative version of the theory where the exclusion axiom is relaxed.

The *Free Energy Principle (FEP)* constitutes a general means of analyzing systems based on the preconditions for their continued existence via implicit models. *Integrated Information Theory (IIT)* provides another general systems theory, focused on what it means for a system to exist from an intrinsic perspective. The extremely broad scope of FEP-AI and IIT suggests (and requires for the sake of conceptual consistency) substantial opportunities for their integration as models of systems and their emergent properties. Within the FEP (and potentially within the scope of IIT), a normative functional-computational account of these modeling processes is suggested in *Active Inference (AI)*. *Hierarchical predictive processing (HPP)* provides an algorithmic and implementational description of means by which systems may minimize prediction error (i.e., free energy) via Bayesian model selection in accordance with FEP-AI. Particular (potentially consciousness-entailing) implementations of HPP have been suggested that involve multi-level modeling via the kinds of architectures suggested by *Global Neuronal Workspace Theory (GNWT)*. The concentric circles depicted above are intended to express increasingly specific modeling approaches with increasingly restricted scopes. (Note: These nesting relations ought not be over-interpreted, as it could be argued that HPP does not require accepting the claims of FEP-AI.) This kind of generative synthesis may potentially be facilitated by developing an additional version of IIT, specifically optimized for analyzing systems without concern for their conscious status, possibly with modified axioms and postulates: *IIT-Consciousness* (i.e., current theory) and *IIT-Emergence* (e.g., alternative formulations that utilize semi-overlapping conceptual-analytic methods). *Integrated World Modeling Theory (IWMT)* distinguishes between phenomenal consciousness (i.e., subjective experience) and conscious access (i.e., higher-order awareness of the contents of consciousness). Non-overlap between the circle containing GNWT and the circle containing IIT-Consciousness is meant to indicate the conceivability of subjectivity-lacking systems that are nonetheless capable of realizing the functional properties of conscious access via workspace architectures. IWMT is agnostic as to whether such systems are actually realizable, either in principle or in practice.

In both cases, IIT would still correspond to an analysis of systems in terms of their irreducible cause–effect power. However, the relaxation of the exclusion axiom in IIT-Emergence could afford a more flexible handling of different kinds of emergent causation (e.g., relative cause–effect power from various coupling systems), as well as more thorough integration with FEP-AI. This broader version of IIT could also sidestep issues such as quasi-panpsychism, as integrated information would not necessarily represent a sufficient condition for generating conscious experiences. While this proposal may not resolve all debates between IIT and GNWT, it may provide further opportunities for integration and synergy between these two theories (e.g., applying—not necessarily consciousness-entailing—phi analyses to posterior and frontal cortices during different stages of cognitive cycles).

### The Bayesian Brain and Hierarchical Predictive Processing (HPP)

Broadly speaking, nervous systems can be straightforwardly understood as generative probabilistic graphical models (PGMs). The directed structure of neurons and their organization into networks of weighted connections generate patterns of effective connectivity (Friston, 1994), where flows of influence are physical instantiations of conditional probabilities. From this perspective, nervous systems can be viewed as modeling the world to the extent neural dynamics reflect patterns in the world. The *Bayesian brain hypothesis* (Friston, 2010) proposes this mutual information takes the form of probabilistic mappings from observations to likely causes, and that these inferences may approach bounded optimality with respect to ecological decision-theoretic objectives (Russell and Subramanian, 1995; Mark et al., 2010; Hoffman and Singh, 2012) over phylogenetic and ontogenetic timescales.

The Bayesian brain hypothesis is supported by evidence for a common cortical algorithm of *hierarchical predictive processing (HPP)*—a potential Rosetta stone for neuroscience (Mumford, 1991; Rao and Ballard, 1999; Hawkins and Blakeslee, 2004). In HPP, neuronal processes constitute hierarchically organized generative models, which attempt to predict likely (hierarchically organized) world states that could have caused actual sensory observations (Friston and Kiebel, 2009; Clark, 2013). Bottom-up sensory information is simultaneously predicted across levels by sending predictions—as Bayesian beliefs, or prior expectations—downwards in anticipation of sensory observations. Prediction-errors (i.e., discrepancies with predictions) are passed upwards toward higher levels, whose modifications update beliefs into posterior expectations, which then become new (empirical) predictions to be passed downwards. This coding scheme is Bayesian in implementing the kind of model selection involved in hierarchical hidden Markov models (George and Hawkins, 2009), or hierarchical Kalman filtering. HPP is also Bayesian in that hierarchical updates combine predictions and prediction-errors according to the relative (estimated) precision of these entailed probability distributions, with this precision-weighting constituting an inverse-temperature parameter by which attention is modulated (Friston et al., 2012b). Notably with respect to the present discussion—and as a source of empirical support for HPP—specific functional roles have been proposed for different frequency bands and cell types, with beta and gamma corresponding to respective predictions and prediction-errors from deep and superficial pyramidal neurons (Bastos et al., 2012; Chao et al., 2018; Scheeringa and Fries, 2019). To summarize, in HPP, each level models the level below it, extending down to sensor and effector systems, with all these models being integrated when they are combined into larger (MB-bounded) generative models (e.g., brains and organisms).

### Generalized HPP and Universal Bayesianism/Darwinism

Although evidence for HPP is strongest with respect to cortex, efficiency considerations (Harrison, 1952) provide reason to believe that this may be a more general phenomenon. Some evidence for extending HPP to non-cortical systems includes decoding of predictive information from retinal cells (Palmer et al., 2015), and also models of motor control involving spinal reflex arcs as predictions (Adams et al., 2013). HPP may further extend beyond nervous system functioning and into processes such as morphogenesis (Friston et al., 2015)—observed to exhibit near-optimal utilization of information (Krotov et al., 2014; Petkova et al., 2019)—and even phylogeny (Ramstead et al., 2018).

This leads to another surprising implication of FEP-AI: the broad applicability of the MB formalism suggests that *any persisting adaptive system will enact some kind of HPP*. More specifically, MB-bound systems contain MB-bound sub-systems, with nesting relations reflecting levels of hierarchical organization. More encompassing (hierarchically higher) models accumulate information from the sub-models they contain, with relative dynamics unfolding on either longer or shorter timescales, depending on relationships among nested MB-bounded systems. The epistemic boundaries instantiated by MBs mean that internal and external states are latent with respect to one another, and so must be inferred. Therefore, the communication of information regarding sub-system internal states (via MBs, definitionally) to the larger systems of which they are part is the propagation of a probabilistic belief—e.g., marginal message passing (Parr et al., 2019)—and so overall hierarchical organization of systems and sub-systems must instantiate HPP.

This generalized HPP may be supported by the near-ubiquitous phenomenon whereby coupling systems minimize free energy more effectively through forming larger systems via mutual entrainment (Jafri et al., 2016). From an FEP-AI perspective, this coupling relationship is one of mutual modeling and collaborative inference (Friston and Frith, 2015; Friston, 2017; Kirchhoff et al., 2018; Palacios et al., 2019). This generalized synchrony (Strogatz, 2012) has also been characterized in thermodynamic terms (Kachman et al., 2017; Friston, 2019), where systems spontaneously self-organize into resonant modes with the environments with which they couple—i.e., absorb work and minimize free energy according to Hamilton's principle of least action—where coordinated dynamics have been observed to contain mutually predictive information (Friston, 2013). Notably, coupled attractors have recently been found to adjust their dynamics beginning at sparsely frequented areas of phase space (Lahav et al., 2018). If these synchronizing manifolds begin to nucleate from improbable (and so surprising) alignments, this flow of (mutual-information maximizing) influence might be functionally understood as updating via “prediction-errors.” While admittedly speculative, these considerations suggest that generalized HPP (and selection for integrated information) could represent a universality class whose potential extensions are nearly as widespread as generalized synchrony itself. Generalized predictive synchrony may also have implications for IIT, potentially helping to explain how internally directed complexes of integrated information can come to resonate with the external world. Further, synchronization dynamics may provide a mechanistic basis for bridging FEP-AI, IIT, and GNWT, as described below with respect to integration via *self-organizing harmonic modes (SOHMs)*.

Free energy may be most effectively minimized—and integrated information maximized (Marshall et al., 2016)—if synchronized couplings take the form of hierarchically organized modules, thus affording robustness, separable optimization, balanced integration and differentiation, evolvability via degeneracy, efficient communication via small-world connectivity, and flexible multi-scale responsivity via critical dynamics (Meunier et al., 2010; Wang et al., 2011; Ódor et al., 2015; Lin and Tegmark, 2017; Lin et al., 2017; Gazzaniga, 2018; Takagi, 2018; Badcock et al., 2019). Hierarchical organization, modularity, and *self-organized criticality (SOC)* may promote both integrated information maximization and free energy minimization (Friston et al., 2012a, 2014; Vázquez-Rodríguez et al., 2017; Hoffmann and Payton, 2018; Salehipour et al., 2018; Khajehabdollahi et al., 2019), potentially suggesting major points of intersection between FEP-AI and IIT across a wide range of systems.

For biological systems, cells integrate information unfolding at cellular scales, with tissues and organs integrating this information at organismic scales, with organisms and groups of organisms integrating this information at even broader scales. It is important to remember that FEP-AI can be viewed as a Bayesian interpretation of *generalized Darwinism* (Kaila and Annila, 2008; Harper, 2011; Frank, 2012; Campbell, 2016), and so these nested couplings can also be viewed in terms of natural selection and niche construction unfolding over multiple hierarchical scales (Constant et al., 2018; Ramstead et al., 2018; Badcock et al., 2019). More specifically, a hierarchy of MBs constitute a hierarchy of selective pressures (Safron, 2019b), with dynamics on one level being selected by the next level of organization. These informational shielding properties of MBs connect with debates regarding units of selection in evolutionary theory, in that only organismic phenotypes—and sometimes groups of organisms (Laland et al., 2015; Richerson et al., 2016)—are “visible” to natural selection with respect to phylogeny. However, specific phenotypes are determined by interactions between internal intrinsic dynamics (i.e., intra-system evolution) as well as external systems with which these dynamics couple via niche construction and phenotypic plasticity (Constant et al., 2018). To the (necessarily limited) extent these adaptively coupled nested scales are shaped by stable selective pressures, then the transmission of information across levels could approach Bayes-optimal (Kaila and Annila, 2008; Payne and Wagner, 2019) active inference by combining all relevant probabilistic influences via gradient ascent/descent over fitness/energy landscapes. That is, what is actively inferred by systems (as generative models) in FEP-AI is the inclusive fitness of the sum-total of all quasi-replicative (i.e., self-evidencing) dynamics capable of interacting on the spatial and temporal scales over which evolution (as inference) occurs.

While this discussion of Bayesian generalized Darwinism may seem needlessly abstract, this multi-level account is essential for understanding what we ought to expect to be generated by competing and cooperating quasi-replicative processes (i.e., evolution). It also provides another potential point of intersection with IIT, in that some dynamics will be more influential than others on the timescales at which interactions occur. Specifically, when considered as networks of relations, some sub-graphs will have more integrated information (i.e., intrinsic cause–effect power, or phi) than others, and phi associated with these subgraphs may parameterize capacity to shape overall directions of evolution.

Importantly, if evolution (as inference) applies not just on the level of phylogeny, but also to intra-organism dynamics, then this provides a means of understanding mental processes as both Bayesian model selection and a kind of (generalized) natural selection (Edelman, 1987). With respect to IIT, the irreducible internal cause-effect power for a particular subnetwork of effective connectivity may correlate with its degree of external cause-effect power in influencing the overall direction of evolution within a mind. If a subnetwork of effective connectivity entails a generative model for enacting particular (adaptive) system–world configurations, then a maximal complex of integrated information would also be a maximally explanatory model for overall system evolution, which may entail consciousness under certain conditions.

In this way, FEP-AI shows how mental causation may be isomorphic with evolutionary causation (i.e., action selection as generalized natural selection), where selective pressures constitute free energy gradients, thus providing formal connections with thermodynamic pressures and power-generation abilities. Power is force integrated over time, which may be more likely to be generated by systems capable of exerting cause–effect power over themselves, suggesting a potentially important role for integrated information in modeling evolutionary dynamics. In this way, by describing mental processes in terms of degrees of self-directed cause–effect power, IIT may help explain how particular processes—including those entailing beliefs and desires—possess varying capacities for contributing to informational and thermodynamic work cycles (Kauffman and Clayton, 2006; Deacon, 2011). Taken together, FEP-AI and IIT show how consciousness may not only represent a system's best guess of what is happening at any given moment, but a source of maximal control energy for system evolution, thus providing a means by which conscious intentions can have causal powers.

While HPP is an extremely broad framework, the difference between *basic active inference* and *adaptive active inference* is important to remember (Kirchhoff et al., 2018): while FEP-AI views all systems *as* models, only some of these models afford adaptivity, and only some systems also *have* models (Seth and Tsakiris, 2018). Living organisms possess specific sub-systems capable of supporting generative models with *temporal depth* and *counterfactual richness* (Friston et al., 2017c). These sub-systems are called brains, and they allow organisms to navigate exchanges with their environments by modeling not just present world configurations, but also possible world configurations predicted based on future (counterfactual) actions (i.e., expected free energy).

Brains acquire especially powerful predictive modeling abilities when they are organized according to multiple layers of hierarchical depth. This deep organization allows these systems to model not only transient events at lower levels, but also their organization into more temporally extended sequences at higher levels (Hawkins and Blakeslee, 2004; Baldassano et al., 2017; Friston et al., 2017c). Further, deep internal dynamics create a potential for functional decoupling between modeling and the unfolding of particular sensorimotor engagements (Tani, 2016; Sormaz et al., 2018; Corcoran et al., 2020), thus enabling counterfactual simulations (Kanai et al., 2019) with temporal “thickness”/“depth” (Humphrey, 2017; Friston, 2018), which when conscious enable imagination and explicit planning. These capacities afford the possibility of constructing rich causal world models (Hassabis and Maguire, 2009; Buchsbaum et al., 2012; Pearl and Mackenzie, 2018; MacKay, 2019), and as discussed below, preconditions for coherent conscious experience. In this way, while all brains may expand autonomous capacity by engaging in HPP, only some architectures may be capable of supporting flexible cognition. Thus, FEP-AI implies a near universality for generative modeling, but not necessarily for consciousness. We will now explore properties of nervous systems that may be particularly important for enabling conscious experiences via complexes of integrated information and global workspaces.

## Mechanisms of Integrated World Modeling

### Self-Organizing Harmonic Modes

IWMT proposes a mechanism by which complexes of integrated information and global workspaces may emerge as metastable synchronous complexes of effective connectivity, or *self-organizing harmonic modes (SOHMs)*. SOHMs are proposed to be attractors and eigenmodes (Friston et al., 2014)—or, solutions to harmonic functions—for phase space descriptions of system dynamics, with particular boundaries depending on network topologies over which synchronization occurs. This view of dynamical systems in terms of SOHMs can be understood as an extension of Atasoy et al.'s (2018) analytical framework wherein spectral decomposition is used to characterize brain activity as mixtures of “*connectome harmonics*.” When this method was first introduced, Atasoy et al. (2016) compellingly demonstrated how reaction-diffusion simulations of spreading activation could generate resting state networks as stable modes—or standing waves—so recapitulating well-known patterns of neuronal organization with minimal assumptions. Intriguingly, hallucinogenic compounds expanded the repertoire of these harmonic modes (Atasoy et al., 2017), increasing spectral diversity and shifting the distribution of modes toward power-law distributions, a putative—albeit controversial (Touboul and Destexhe, 2017)—hallmark of criticality (Fontenele et al., 2019). This finding is consistent with other studies of psychedelic compounds (Tagliazucchi et al., 2014; Schartner et al., 2017; Viol et al., 2017), supporting the hypothesis that brains may enhance dynamical reconfigurability by being “tuned” toward near-critical regimes (Pletzer et al., 2010; Haimovici et al., 2013; Carhart-Harris, 2018).

Atasoy et al. (2016) describe this modeling approach of identifying eigenfunctions (over a system's Laplacian) as having an extremely broad scope, with applications ranging from Turing's (1952) account of morphogenesis, to acoustic phenomena and other patterns observed with vibrating media (Ullmann, 2007), to solutions for electron orbitals in quantum mechanics (Schrödinger, 1926). Based on our previous discussion of probabilistic graphical models as a near-universal representational framework, the term “connectome harmonics” could be reasonably generalized to apply to all systems. However, IWMT introduces the new term of “SOHMs” to prevent confusion and to emphasize the dynamic self-organizing processes by which synchronous complexes may emerge, even when constituting local standing wave descriptions over dynamics (rather than constituting a Fourier basis for an entire connected system). That is, Atasoy's connectome harmonics constitute a more specific—and important for the sake of understanding consciousness—variety of SOHM.

SOHMs may act as systemic causes in selecting specific dynamics through synchronous signal amplification, with micro-dynamics having greater contributions to synchronizing macro-dynamics when phase-aligned. SOHMs could be viewed as either standing or traveling waves, depending on the level of granularity with which they are modeled (Friston et al., 2014; Mišić et al., 2015; Atasoy et al., 2018; Muller et al., 2018; Zhang et al., 2018). However, when viewed as harmonic modes, SOHMs would have specific boundaries and timescales of formation. In this way, resonant signal amplification within SOHMs could select patterns of effective connectivity based on the timescales at which maximal coherence is achieved. IWMT specifically proposes that these synchronous complexes promote “*communication through coherence*” (Hebb, 1949; Dehaene, 2014; Fries, 2015; Deco and Kringelbach, 2016; Hahn et al., 2019). From an FEP-AI perspective, this synchrony-enhanced communication would facilitate information sharing among (and marginalization over) coupled dynamics, thereby organizing message passing (or belief propagation) for inference (Parr and Friston, 2018a; Parr et al., 2019).

With respect to emergent causation, *circular causal processes* by which SOHMs form would constitute organization into renormalization groups and attracting flow paths along center manifolds (Haken, 1977, 1992; Bogolyubov and Shirkov, 1980; Li and Wang, 2018; Shine et al., 2019). This synchronization of micro-scale phenomena into larger groupings on meso- and macro-scales could be viewed as a kind of informational closure and coarse-graining (Hoel et al., 2016; Chang et al., 2019). Further, for self-evidencing generative models (Hohwy, 2016; Yufik and Friston, 2016; Kirchhoff et al., 2018), integrating processes underlying SOHM formation would calculate marginal joint posteriors based on specific (Bayesian) beliefs entailed by particular patterns of effective connectivity within and between various synchronous complexes.

*[Note: More details on potential mechanisms for SOHM formation and functional consequences can be found in IWMT Revisited* (Safron, 2019a) *in the sections: “Phenomenal binding via ESMs (Embodied Self-Models)” and “Mechanisms for integration and workspace dynamics.”]*

### SOHMs as Dynamic Cores of Integrated Information and Workspaces

With respect to conscious perception, the resonant signal amplification by which SOHMs emerge could potentially contribute to the calculation of highly precise—albeit not necessarily accurate (Hohwy, 2012; Vul et al., 2014)—joint distributions (or maximal a posteriori (MAP) estimates derived thereof). The ability of synchronous complexes to select phase-aligned patterns has clear correspondences with theories of consciousness emphasizing re-entrant signaling (Singer, 2001; Varela et al., 2001; Crick and Koch, 2003; Edelman et al., 2011; Shanahan, 2012; Dehaene, 2014; Grossberg, 2017) and in terms of Bayesian model selection (Hohwy, 2012, 2013), could be understood as promoting winner-take-all dynamics among competing and cooperating inferential flows. SOHMs may also help provide mechanistic bases for “ignition” events accompanying phase transitions in which perception becomes conscious (Dehaene and Changeux, 2011; Friston et al., 2012a; Arese Lucini et al., 2019). IWMT specifically proposes that conscious ignition corresponds to surpassing critical thresholds for SOHM formation via self-synchronized neural activity, thus forming meta-stable complexes as dynamic cores of integrated information, functioning as neuronal global workspaces.

The ability of SOHMs to select aligned patterns may help explain how seemingly definite experiences could emerge from probabilistic world models (Wiese, 2017; Block, 2018; Clark, 2018; Gross, 2018), as opposed to generating a “Bayesian blur,” or superposition of possibilities. This hypothesis is consistent with Clark's (2018) suggestion that coherent and precise inference stems from requirements for engaging with environments via sensorimotor couplings (Clark, 2016). Along these lines, by enabling the generation of inferences with rapidity and reliability, SOHMs could afford approximate models capable of guiding action–perception cycles and decision-making (von Uexküll, 1957; Fuster, 2009; Madl et al., 2011; Vul et al., 2014; Linson et al., 2018; Parr and Friston, 2018b). Further, these sensorimotor engagements may promote SOHM formation by providing coherent sources of correlated information, thus affording the possibility of learning even more sophisticated models (Pfeifer and Bongard, 2006; Safron, 2019a,c). IWMT proposes that this continual shaping of behavior based on rich causal world models may be both a major adaptive function of consciousness and a precondition for developing coherent conscious experience. [Note: If consciousness requires semiotic closure Chang et al., 2019 via action–perception cycles, then this cybernetic grounding suggests that systems like plants and insect colonies are unlikely to be conscious, even if capable of sophisticated (but limited) levels of intelligence.]

SOHM dynamics may help to explain many kinds of rhythmic phenomena, such as the fact that oscillations tend to occur at faster rates in organisms with smaller brains (Buzsáki and Watson, 2012); all else being equal, smaller systems are likely to arrive at synchronous equilibria more quickly, with larger systems requiring relatively more time for synchronizing their micro-dynamics. SOHMs may also help to explain why different rhythms (Table 3) would be associated with different processes in hierarchical predictive processing (HPP) (Bastos et al., 2015; Sedley et al., 2016; Chao et al., 2018), where faster gamma oscillations communicate bottom-up prediction-errors ‘calculated' by local microcircuits, and where slower beta oscillations generate top-down predictions via integrating information (i.e., accumulating model evidence) from more spatially-extended sources. These beta complexes may potentially be organized via nesting within even larger and slower-forming SOHMs, such as those generated at alpha, theta, and delta frequencies. This cross-frequency phase coupling (Canolty and Knight, 2010) could allow for the stabilization of multi-scale dynamics within HPP, with increasing levels of hierarchical depth affording modeling of complex and temporally extended causes (Friston et al., 2017c). Hierarchical nesting of SOHMs could allow modeling to simultaneously (and synergistically) occur at multiple levels of granularity, thus affording both global stability (Humphrey, 2017) and fine-grained adaptive control as overall systems couple with their environments.

**Table 3:** Neural frequency bands, their potential roles in predictive processing, and possible experiential consequences.

**Frequency band**	**Role in predictive processing**	**Potential experiential consequences**
Gamma (~30–120 Hz)	Ascending prediction-errors	Sensory sensitivity and detail
Beta (~13–30 Hz)	Descending predictions	Perceptual vividness
Alpha (~8–12 Hz)	Predictions integrated into coherent (egocentric) spatial, temporal, and causal reference frames	Basic phenomenal consciousness
Theta (~3–7 Hz)	Predictions integrated with internally-generated actions and comparisons among recent (and counterfactual) experiences	Access consciousness, agency, and shaping of phenomenal consciousness via actions
Delta (~0.5–2 Hz)	Higher-level predictions for active inference unfolding at slower and more inclusive temporal and spatial scales	Unclear; possibly autonoetic consciousness and complex cognition; emotions and feelings, broadly construed as global alterations of states of consciousness and means of aligning spatiotemporal dynamics between mind and world (Northoff and Huang, 2017)

If SOHMs integrate information in the ways suggested here—marginalizing over synchronized components—then the largest SOHM of a system would generate a joint posterior (or estimate derived thereof) over all smaller SOHMs contained within its scope. These encompassing SOHMs would integrate information across heterogeneous processes, as well as affording unified sources of control energy for system evolution. These maximal SOHMs could generate estimates of overall organismic states, thus forming dynamic cores of integration for perception and action, potentially enabling autonomous control by integrated self-processes. Further, privileged positions of maximal SOHMs with respect to network centrality (Aadithya et al., 2010) and modeling capacity could promote directional entrainment of smaller complexes, thus promoting coherent agentic action selection.

For biological systems, the dynamics within maximal SOHMs may have the clearest correspondences with events unfolding at organismic scales. For organisms such as *C. elegans*, these dynamics might unfold at the frequencies of locomotory eigenmodes, potentially concentrated in a core of richly connected nodes (Towlson et al., 2013), thus allowing enslavement of a worm's peripheral pattern generators by predictive models coordinating the enaction of coherent movement vectors. For organisms such as *Homo sapiens*, these dynamics might unfold at the frequencies of real and imagined sensorimotor contingencies (Elton, 2000; O'Regan and Noë, 2001; Tani, 2016; Chen et al., 2017; Prinz, 2017; Zadbood et al., 2017; Baldassano et al., 2018; Chang et al., 2019), potentially concentrated along deep portions of cortical generative models, thus allowing enslavement of an individual's sensorium and effectors by rich causal models of self and world. Whether in worms or humans, SOHMs would entail joint posteriors (or associated maximal estimates) from probabilistic models for embodied agents and the environments with which they couple. In these ways, Maximal SOHMs may be coextensive with both maxima of integrated information (i.e., MICS) and global workspaces. However, while SOHMs with the greatest amount of irreducible integrated information may correspond to basic phenomenal consciousness (e.g., complexes centered on posterior cortices), organization into an even larger (albeit possibly less irreducibly integrated) synchronous complex involving the frontal lobes may be required for access consciousness and agentic control.

A multi-level understanding of SOHMs in terms of neuronal dynamics and probabilistic inference suggests that we should expect these complexes to form over subnetworks with coherent mutual information, which is more likely if patterns of effective connectivity entail coherent and well-evidenced world models. With respect to loopy message passing for approximate inference (Koller and Friedman, 2009; Friston et al., 2017b), these coherent models may have a (circular) causal significance in that they would be more likely to provide consistent inferential flows, and so be more likely to first converge upon stable posteriors, and so be more likely to dominate rounds of Bayesian model selection. Notably, this kind of convergence is more likely for Bayesian networks that balance integration and differentiation—associated with high phi (Marshall et al., 2016)—and this is precisely what is observed for “rich club” connectivity cores (Sporns, 2013; Mišić et al., 2015; Cohen and D'Esposito, 2016; Mohr et al., 2016). Further, high degrees of re-entrant connectivity and potential for recurrent dynamics suggests that these richly connected networks are particularly likely to serve as loci of “ignition” events in global workspace models (Dehaene and Changeux, 2011; Shanahan, 2012). Finally, considering that integrated information reflects a system's ability to exert cause–effect power over itself, SOHMs may be particularly likely to form along high phi networks.

### IWMT and Maximizing SOHMs: Bringing Forth Worlds of Experience

A maximal SOHM—as a MICS and MICE repertoires—within a brain may center on posterior cortices, and in particular the temporoparietal junction (Graziano, 2019) and posteromedial cortices (PMCs) (O'Reilly et al., 2017), with synchronizing complexes forming at alpha frequencies generating basic phenomenal consciousness. Nesting of these alpha rhythms within theta frequencies may further allow for coupling with the frontal lobes and hippocampal complex, thus affording goal-directed and access consciousness from global workspace dynamics. IWMT's focus on PMCs and alpha frequencies (as synchronizing manifolds) is based on both the types of information available to these systems/processes (Papez, 1937; Jann et al., 2009; Gramann et al., 2010; Knyazev et al., 2011; Damasio, 2012), as well as empirical associations with attention and working memory (Palva and Palva, 2011; Kerr et al., 2013; Michalareas et al., 2016; Sato et al., 2018; Bagherzadeh et al., 2019). PMCs receive information from upper levels of each sensory hierarchy, as well as the position of an organism in space, including head-direction information. This information is likely a prerequisite for organizing perception into egocentric reference frames (Brewer et al., 2011, 2013; Guterstam et al., 2015; Li et al., 2018; Smigielski et al., 2019). In line with models in which consciousness depends on projective geometry (Rudrauf et al., 2017; Williford et al., 2018), a stable source of egocentric perspective may represent a practically necessary precondition for there to be “something that it is like:” i.e., the ability to generate models with spatial, temporal, and causal coherence for system and world.

IWMT focuses on space, time (i.e., relative dynamics in space), and cause (i.e., predictable regularities in these dynamics), but wholistic self-processes (Damasio, 2012; Humphrey, 2017) may also be essential for developing world models capable of generating coherent subjectivity. Self-processes may be practically necessary for consciousness because the integration of large-scale brain activity may be required for the coherent regulation of action–perception cycles, and thereby cybernetic sense-making. Self-processes could allow for selection of specific models on the basis of relevance (Shanahan and Baars, 2005; Davey and Harrison, 2018; Linson et al., 2018; Hattori et al., 2019), with stable self-models extending this organization across time (Dennett, 1992; Hirsh et al., 2013; Buonomano, 2017), thereby enabling the learning required to construct experienceable world models. In brief, IWMT proposes that Kant's preconditions for judgment are also necessary preconditions for consciousness (Northoff, 2012; De Kock, 2016). While PMCs may be sufficient for basic phenomenal consciousness, larger complexes may be required for certain kinds of higher-order cognition, including access and autonoetic consciousness (Brown et al., 2019; LeDoux, 2019; Shea and Frith, 2019). This integration of action with perception is likely crucial for agentic planning and the counterfactual simulations upon which it is based (Hassabis and Maguire, 2009; MacKay, 2019), without which the development of coherent world models may be impossible (De Kock, 2016; Friston, 2017).

To summarize (Table 4), in systems where synchrony both emerges from and facilitates coherent message passing, SOHMs enable both workspace dynamics and high degrees of meaningful informational integration, where meaning is a difference that makes a difference to the ability of a system to survive and achieve its goals. However, integrated information and workspaces only entail consciousness when applied to systems that can also be understood as Bayesian belief networks, where beliefs have coherence because they have actual semantic content by virtue of evolving through interactions with a coherently structured (and so semi-predictable) world. Without those meaningful external connections, systems could have arbitrarily large amounts of integrative potential, but there still may be nothing that it is like to be such systems.

**Table 4:** Integrating IIT with the FEP-AI framework and IWMT's model of communication through coherence via SOHM dynamics.

**Integrated Information Theory (IIT) axioms and postulates**	**Integration with the Free Energy Principle and Active Inference (FEP-AI) Framework**	**Integration via Self-Organizing Harmonic Modes (SOHMs): Eigenmodes of effective connectivity and synchronization manifolds**
**Intrinsic existence:**		
Systems exert C–E power on themselves and the sub-systems of which they are composed. Sub-systems exert C–E power on themselves and the larger systems of which they are a part. C–E power exists at particular spatial and temporal grains.	Systems are describable as PGMs, where graphs express conditional dependence structure between sub-components. All systems and sub-systems possess defining MBs, the boundaries of which establish conditional independence between internal and external states. MB internal states can only interact with themselves, or with external states via MBs. Persisting systems preserve their MBs by exerting C–E power both on themselves and other systems.	SOHMs (and their MB boundaries) form as systems and sub-systems interact with both themselves and other systems at particular spatial and temporal grains. SOHMs influence how systems as wholes are likely to interact with both themselves and other systems at varying levels of granularity. SOHMs are both consequences and causes of the processes that generate them, both emerging from and determining the C–E power that systems exert on themselves and other systems.
**Composition:**		
Systems are composed of sub-systems with particular inter-relations. Structured inter-relations determine the specific C–E power of systems on sub-systems, which exert C–E power on each other.	PGMs are composed of connected elements with particular components differentially contributing to joint probability distributions. Graph structures define relations of conditional dependence and independence, so determining inferential flows within and between MBs (i.e., marginalization and message passing). Persisting MB compositions are generative models for those particular compositions.	Particular system compositions influence the dynamics of SOHM formation, which, in turn, influence patterns of effective connectivity between and within system sub-components. Subnetworks along which SOHMs form determine how C–E power flows on different timescales, including with respect to SOHM formation processes. SOHMs have specific spatial and temporal extents, so defining systems and sub-systems in terms of particular inter-relations.
**Information:**		
Systems have specific compositions that are differentiated from other possible compositions. C–E repertoire: probability distribution over all permutations of possible causes and effects that a system could exert on itself.	MB-defined dependency relations specify inferential properties of PGMs, including probability distributions and estimates for likely causes of present observations, given past observations. Mappings from observations to likely causes define systems as generative models.	Specific combinations of SOHMs and their particular compositions influence (and are influenced by) effective connectivity within and between systems, so specifying the particular information content of those systems. By promoting communication through coherence, MB-bounded SOHMs can implement marginalization over sub-networks and organize message passing and/or belief propagation.
**Conceptual structure:**		
Mapping of C–E repertoires onto an abstract metric space, specifying particular causal properties.	Persisting systems generate themselves as particular densities, so providing mutual information between past and future states, and between internal and external states of MB-bound systems.	Different systems will have different SOHMs, so generating inferences that are differentiated from other systems in which different groups of elements would be included within synchronizing manifolds.
**Integration:**		
Systems are unified in terms of being irreducible to independent subcomponents. This irreducibility can be quantified (phi) by comparing C–E repertoires before and after systems are divided by a minimally disruptive partitioning, known as a “minimal information partition” (MIP).	All components of MB-bounded sub-graphs from PGMs (differentially) contribute to integrating—literally, calculating integrals for—associated marginal joint probability distributions. Persisting systems are unified (to varying degrees); all components contribute to self-evidencing (to varying extents). By quantifying the integrated complexity of system-internal C–E power, the phi of an MB-bound set will correlate with the marginal likelihood (or negative free energy) associated with particular self-evidencing systems.	SOHMs are unified (to varying degrees); all components of self-interacting systems contribute (to varying extents) to the emergence of its particular eigenmodes. If SOHMs influence and are influenced by the particular configuration of a system, then any alteration will result in different patterns of effective connectivity. If SOHMs promote information transmission, then any SOHM modification will change inferences, where the least of these alterations would constitute a MIP.
**Exclusion:**		
Systems have definite boundaries with respect to their ability to exert C–E power over particular spatial and temporal grains. IIT identifies intrinsically existing systems as complexes, specifying maximally irreducible conceptual structures (MICS) and associated maximally irreducible cause-effect (MICE) repertoires.	PGMs represent multiple possibilities, but they can also generate precise posterior distributions and discrete estimates of likely parameter values. Larger systems can integrate marginal probabilities from MB-bounded sub-systems, so integrating more information into models. If phi promotes self-generation, then boundaries for maximal complexes would correspond to boundaries for (free-energy-minimizing) systems generating maximal self-model evidence, with maximal potential influences on overall system evolution.	The specific temporal and spatial scales governing SOHM formation will constrain opportunities for influencing the evolution of these self-synchronizing attracting manifolds. The MB boundaries of SOHMs will define which dynamics are capable of contributing to joint inference to which degrees. Theoretically, rapidly forming and strongly synchronizing SOHMs could entail precise joint probabilities, or maximum a posteriori (MAP) estimates derived thereof.

*C-E, Cause-effect; PGM, Probabilistic graphical model; MB, Markov blanket*.

*[Note: For some testable hypotheses related to these ideas, please refer to* *Supplementary Material**.]*

## Discussion: Toward Solving the Enduring Problems of Consciousness (and AI?)

*[Note: More details on computational principles and systems likely to be associated with consciousness can be found in IWMT Revisited* (Safron, 2019a) *in the sections, “Machine learning architectures and predictive processing models of brain and mind” and “Consciousness: Here, There, but Not Everywhere.”]*

### Autoencoders, Predictive Processing, and the Conscious Turbo Code

Helmholtz (1878) is often viewed as providing the first clear description of perception as inference:

“*Objects are always imagined as being present in the field of vision as would have to be there in order to produce the same impression on the nervous mechanism*.”

Dayan, Hinton, Neal, and Zemel (Dayan et al., 1995) constructed machine learning systems based on these principles, trained using cost functions based on Helmholtz free energy. These kinds of architectures can be trained to handle noisy inputs or infer missing data, with more recent versions being able to generate completely novel combinations of features. These are all aspects of conscious (and unconscious) perception and have many commonalities with HPP within FEP-AI.

**Figure 2:**
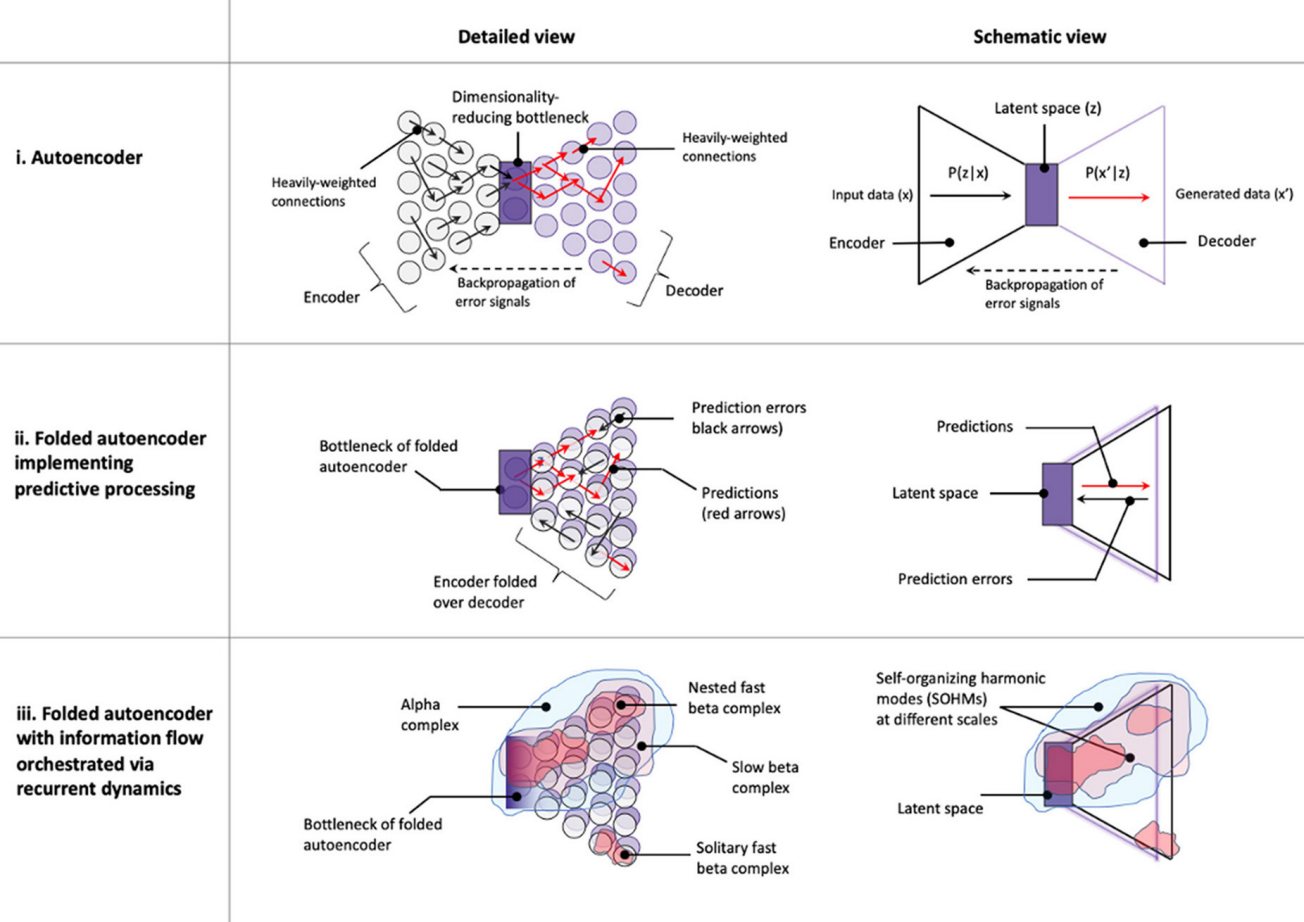
Sparse folded variational autoencoders with recurrent dynamics via self-organizing harmonic modes (SOHMs).

Variational autoencoders (Kingma and Welling, 2014) are composed of encoders and generative decoders connected by low-dimensional bottlenecks, where encoders learn to compress input data into reduced-dimensionality feature spaces, and where decoders learn to use these latent features to infer likely details of higher-dimensional data. HPP models of sensory cortices (Figure 2; Table 5) may be approximated as disentangled variational autoencoders, where encoders and decoders are constituted by respective hierarchies of superficial and deep pyramidal neurons (Kanai et al., 2019). However, rather than training solely based on divergences between respective input and output layers of encoder and decoder networks, prediction-error is minimized at all levels simultaneously based on comparisons between time-varying sensory observations and internally-generated predictions. HPP in brains further involves multiple interacting autoencoding hierarchies, with connections being particularly strong in deeper association cortices—corresponding to reduced dimensionality latent spaces—thus affording synergistic inferential power with shared priors from multi-modal sensory integration and world modeling.

**(i) Autoencoder**.

An autoencoder is a type of artificial neural network that learns efficient representations of data, potentially including a capacity for generating more complete data from less complete sources. The encoder compresses input data over stages of hierarchical feature extraction, passes it through a dimensionality-reducing bottleneck and into a decoder. The decoder attempts to generate a representation of the input data from these reduced-dimensionality latent representations. Through backpropagation of error signals, connections contributing to a more inaccurate representation are less heavily weighted. With training, the decoder learns how to generate increasingly high-fidelity data by utilizing the compressed (and potentially interpretable) feature representations encoded in the latent space of the bottleneck portion of the network. In the more detailed view on the left, black arrows on the encoder side represent connections contributing to relatively high marginal likelihoods for particular latent feature space representations, given connection weights and data. Red arrows on the decoder side represent connections with relatively high marginal likelihoods for those reconstructed features, given connection weights and latent space feature hypotheses. While these autoencoders are fully connected dense networks, particular connections are depicted (and associated probabilities discussed) because of their relevance for predictive processing. Note: Although the language of probability theory is being used here to connect with neurobiologically-inspired implementations, this probabilistic interpretation—and links to brain functioning—is more commonly associated with variational autoencoders, which divide latent spaces into mean and variance distributions parameterized by stochastic sampling operations in generating likely patterns of data, given experience.

**(ii) Folded autoencoder implementing predictive processing**.

In this implementation of predictive processing, autoencoders are ‘folded' at their low-dimensionality bottlenecks—such that corresponding encoding and decoding layers are aligned—with decoding hierarchies (purple circles) depicted as positioned underneath encoding hierarchies (gray circles). Within a brain, these decoding and encoding hierarchies may correspond to respective populations of deep and superficial pyramidal neurons (Bastos et al., 2012). In the figure, individual nodes represent either units in an artificial network—or groups of units; e.g., capsule networks (Kosiorek et al., 2019)—or neurons (or neuronal groups; e.g., cortical minicolumns) in a brain. Predictions (red arrows) suppress input signals when successfully predicted, and are depicted as traveling downwards from representational bottlenecks (corresponding to latent spaces) along which autoencoding networks are folded. Prediction errors, or observations for a given level (black arrows) continue to travel upwards through encoders unless they are successfully predicted, and so “explained away.” Data observations (i.e., prediction errors) are depicted as being sparser relative to high-weight connections in the (non-folded) encoding network presented above, where sparsity is induced via predictive suppression of ascending signals. This information flow may also be viewed as Bayesian belief propagation or (marginal) message passing (Friston et al., 2017b; Parr et al., 2019). In contrast to variational autoencoders in which training proceeds via backpropagation with separable forward and backward passes—where cost functions both minimize reconstruction loss and deviations between posterior latent distributions and priors (usually taking the form of a unit Gaussian)—training is suggested to occur (largely) continuously in predictive processing (via folded autoencoders), similarly to recent proposals of target propagation (Hinton, 2017; Lillicrap et al., 2020). Note: Folded autoencoders could potentially be elaborated to include attention mechanisms, wherein higher-level nodes may increase the information gain on ascending prediction-errors, corresponding to precision-weighting (i.e., inverse variance over implicit Bayesian beliefs) over selected feature representations.

**(iii) Folded autoencoder with information flows orchestrated via recurrent dynamics**.

This row shows a folded autoencoder model of a cortical hierarchy, wherein neuronal oscillations mediate predictions—potentially orchestrated by deep pyramidal neurons and thalamic (and striatal) relays—here characterized as self-organizing harmonic modes (SOHMs). This paper introduces SOHMs as mechanisms realizingsynchronization manifolds for coupling neural systems (Palacios et al., 2019), and sources of coherent neuronal oscillations and evidence accumulation for predictive processing. Depending on the level of granularity being considered, these predictive oscillations could either be viewed as traveling or standing waves (i.e., harmonics). SOHM-based predictions are shown as beta oscillations forming multiple spatial and temporal scales. These predictive waves may be particularly likely to originate from hierarchically higher levels—corresponding to latent spaces of representational bottlenecks—potentially due to a relatively greater amount of internal reciprocal connectivity, consistent information due to information aggregation, or both. SOHMs may also occur at hierarchically lower levels due to a critical mass of model evidence accumulation allowing for the generation of coherent local predictions, or potentially on account of semi-stochastic synchronization. Faster and smaller beta complexes are depicted as nested within a larger and slower beta complex, all of which are nested within a relatively larger and slower alpha complex. Note: In contrast to standard machine learning implementations, for this proposal of predictive processing via folded autoencoders (and SOHMs), latent space is depicted as having unclear boundaries due to its realization via recurrent dynamics. Further, inverse relationships between the spatial extent and speed of formation for SOHMs are suggested due to the relative difficulties of converging on synchronous dynamics within systems of various sizes; theoretically, this mechanism could allow for hierarchical modeling of events in the world for which smaller dynamics would be expected to change more quickly, and where larger dynamics would be expected to change more slowly.

**Table 5:** Proposed correspondences between features of variational autoencoders and predictive processing.

**Variational autoencoder features**	**Proposed correspondences in predictive processing**
Encoder network	Ascending hierarchy of superficial pyramidal neurons; Message-passing at gamma frequencies
Generative decoder network	Descending hierarchy of deep pyramidal neurons; Beliefs propagated at beta frequencies
Reduced dimensionality bottleneck	Association cortices and deeper portions of generative models; Estimates calculated at beta, alpha, and theta frequencies
Mean vectors	Activity levels for neuronal populations at different parts of hierarchy
Variance vectors	Neuronal population activity variability
Sampling from latent feature space	Large-scale synchronous complexes at beta, alpha, and theta frequencies; “ignition” events
Training: minimizing reconstruction loss between input layer of encoder and output layer of generative decoder; also minimizing divergence from unit Gaussian, parameterized by disentangling parameter	Training: minimizing precision-weighted prediction-errors at all layers simultaneously; precision-weighting as analogous to disentanglement hyperparameter; many mechanisms including synchronous gain control and diffuse neuromodulatory systems
Potential for sequential organization via recurrent network controllers (Ha and Schmidhuber, 2018)	Organization of state transitions by hippocampal system and frontal cortices (Koster et al., 2018)

**Figure 3:**
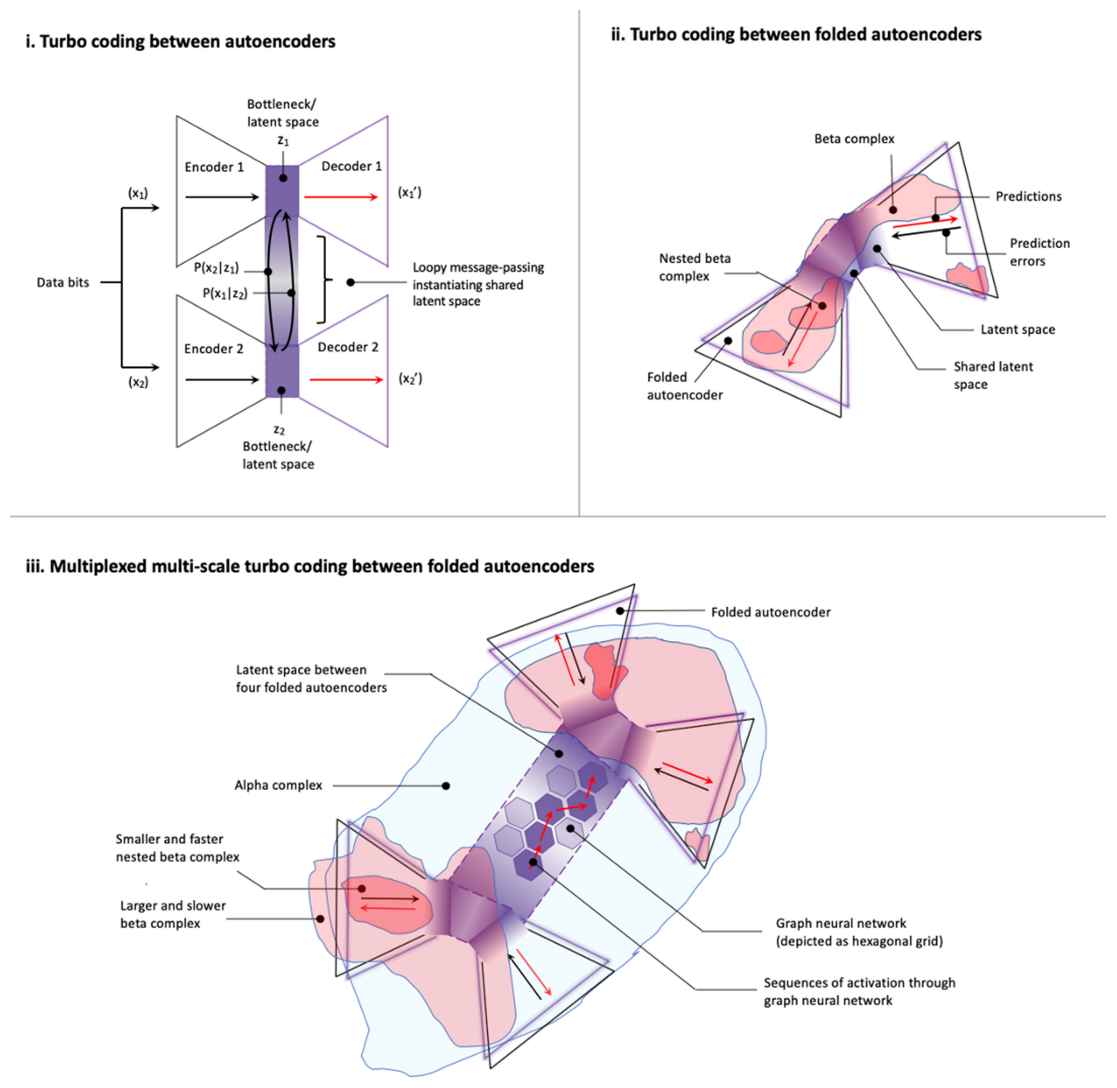
Cortical turbo codes.

IWMT proposes that connections between the low-dimensionality bottlenecks from various modalities may form an auto-associative network supporting loopy belief propagation—or message passing—thus constituting a turbo code (Berrou and Glavieux, 1996), and hence approaching the Shannon limit with respect to optimality in communicating information over noisy channels (Figure 3; Table 6). [Note: While any instantiation of loopy belief propagation may be understood as realizing a turbo code, IWMT specifically suggests that a broad network of cross-modal effective connectivity is required for coherent integrated world modeling.] This framing of HPP in terms of autoencoders and turbo codes could provide a computational analog for neural systems underlying consciousness: a reduced-dimensionality representational bottleneck that extracts the most important details from sensory data, and which affords inferential synergy by providing a workspace where specialist models can be combined, integrated, and then rebroadcast. [Note: HPP dimensionality-reduction may have relevance to the sketch-like nature of awareness proposed in Graziano's Attention Schema Theory (Graziano, 2013, 2019).] According to IWMT, coherent self-world modeling likely also requires organizing this information into spatiotemporal trajectories, as afforded by the hippocampal system and machine learning architectures that attempt to reproduce its functioning (Fraccaro et al., 2017; Ha and Schmidhuber, 2018; Whittington et al., 2018; Wu et al., 2018), and as suggested by impaired counterfactual modeling with medial temporal lobe damage (Hassabis and Maguire, 2009; MacKay, 2019).

**(i) Turbo coding between autoencoders**.

Turbo coding allows signals to be transmitted over noisy channels with high fidelity, approaching the theoretical optimum of the Shannon limit. Data bits are distributed across two encoders, which compress signals as they are passed through a dimensionality reducing bottleneck—constituting a noisy channel—and are then passed through decoders to be reconstructed. To represent the original data source from compressed signals, bottlenecks communicate information about their respective (noisy) bits via loopy message passing. Bottleneck z_1_ calculates a posterior over its input data, which is now passed to Bottleneck z_2_ as a prior for inferring a likely reconstruction (or posterior) over its data. This posterior is then passed back in the other direction (Bottleneck z_2_ to Bottleneck z_1_) as a new prior over its input data, which will then be used to infer a new posterior distribution. This iterative Bayesian updating repeats multiple times until bottlenecks converge on stable joint posteriors over their respective (now less noisy) bits. IWMT proposes that this operation corresponds to the formation of synchronous complexes as self-organizing harmonic modes (SOHMs), entailing marginalization over synchronized subnetworks—and/or precision-weighting of effectively connected representations—with some SOHM-formation events corresponding to conscious “ignition” as described in Global Neuronal Workspace Theory (Dehaene, 2014). However, this process is proposed to provide a means of efficiently realizing (discretely updated) multi-modal sensory integration, regardless of whether “global availability” is involved. Theoretically, this setup could allow for greater data efficiency with respect to achieving inferential synergy and minimizing reconstruction loss during training in both biological and artificial systems. In terms of concepts from variational autoencoders, this loopy message passing over bottlenecks is proposed to entail discrete updating and maximal a posteriori (MAP) estimates, which are used to parameterize semi-stochastic sampling operations by decoders, so enabling the iterative generation of likely patterns of data, given past experience (i.e., training) and present context (i.e., recent data preceding turbo coding). Note: In turbo coding as used in industrial applications such as enhanced telecommunications, loopy message passing usually proceeds between interlaced decoder networks; within cortex, turbo coding could potentially occur with multiple (potentially nested) intermediate stages in deep cortical hierarchies.

**(ii) Turbo coding between folded autoencoders**.

This panel shows turbo coding between two folded autoencoders connected by a shared latent space. Each folded autoencoder sends predictions downwards from its bottleneck (entailing reduced-dimensionality latent spaces), and sends prediction errors upwards from its inputs. These coupled folded autoencoders constitute a turbo code by engaging in loopy message passing, which when realized via coupled representational bottlenecks is depicted as instantiating a shared latent space via high-bandwidth effective connectivity. Latent spaces are depicted as having unclear boundaries—indicated by shaded gradients—due to their semi-stochastic realization via the recurrent dynamics. A synchronous beta complex is depicted as centered on the bottleneck latent space—along which encoding and decoding networks are folded—and spreading into autoencoding hierarchies. In neural systems, this spreading belief propagation (or message-passing) may take the form of traveling waves of predictions, which are here understood as self-organizing harmonic modes (SOHMs) when coarse-grained as standing waves and synchronization manifolds for coupling neural systems. Relatively smaller and faster beta complexes are depicted as nested within—and potentially cross-frequency phase coupled by—this larger and slower beta complex. This kind of nesting may potentially afford multi-scale representational hierarchies of varying degrees of spatial and temporal granularity for modeling multi-scale world dynamics. An isolated (small and fast) beta complex is depicted as emerging outside of the larger (and slower) beta complex originating from hierarchically higher subnetworks (hosting shared latent space). All SOHMs may be understood as instances of turbo coding, parameterizing generative hierarchies via marginal maximum a posteriori (MAP) estimates from the subnetworks within their scope. However, unless these smaller SOHMs are functionally nested within larger SOHMs, they will be limited in their ability to both inform and be informed by larger zones of integration (as probabilistic inference).

**(iii) Multiplexed multi-scale turbo coding between folded autoencoders**.

This panel shows turbo coding between four folded autoencoders. These folded autoencoders are depicted as engaging in turbo coding via loopy message passing, instantiated by self-organizing harmonic modes (SOHMs) (as beta complexes, in pink), so forming shared latent spaces. Turbo coding is further depicted as taking place between all four folded autoencoders (via an alpha complex, in blue), so instantiating further (hierarchical) turbo coding and thereby a larger shared latent space, so enabling predictive modeling of causes that achieve coherence via larger (and more slowly forming) modes of informational integration. This shared latent space is illustrated as containing an embedded graph neural network (GNN) (Liu et al., 2019; Steppa and Holch, 2019), depicted as a hexagonal grid, as a means of integrating information via structured representations, where resulting predictions can then be propagated downward to individual folded autoencoders. Variable shading within the hexagonal grid-space of the GNN is meant to indicate degrees of recurrent activity—potentially implementing further turbo coding—and red arrows over this grid are meant to indicate sequences of activation, and potentially representations of trajectories through feature spaces. These graph-grid structured representational spaces may also afford reference frames at various levels of abstraction; e.g., space proper, degrees of locality with respect to semantic distance, abductive connections between symbols, causal relations, etc. If these (alpha- and beta-synchronized) structured representational dynamics and associated predictions afford world models with spatial, temporal, and causal coherence, these processes may entail phenomenal consciousness. Even larger integrative SOHMs may tend to center on long-distance white matter bundles establishing a core subnetwork of neuronal hubs with rich-club connectivity (van den Heuvel and Sporns, 2011). If hippocampal-parietal synchronization is established (typically at theta frequencies), then bidirectional pointers between neocortex and the entorhinal system may allow decoders to generate likely patterns of data according to trajectories of the overall system through space and time, potentially enabling episodic memory and imagination. If frontal-parietal synchronization is established (potentially involving theta-, alpha-, and beta- synchrony), these larger SOHMs may also correspond to “ignition” events as normally understood in Global Neuronal Workspace Theory, potentially entailing access consciousness and volitional control.

**Table 6:** Proposed correspondences between turbo coding in artificial neural networks and biological neural dynamics.

**Turbo codes in artificial neural networks**	**Proposed correspondences in brains**
Take data and distribute bits over two encoder–decoder networks.	Each sensory modality can be modeled as a noisy channel.
Generate a posterior probability estimate of the signal in one of the networks.	Within modalities, bottom-up updated states of deeper hierarchical levels calculate local posteriors (possibly taking the form of locally synchronized fast beta complexes).
Take the posterior from this network and propagate that belief as a prior to inform the calculation of a joint posterior for the other network.	Between modalities, auto-associative linkages across deeper hierarchical levels allow posteriors to be shared as empirical priors (possibly taking the form of larger and slower beta complexes).
Pass this message back to the original network as priors to inform the calculation of a new posterior.	Modalities are likely to be reciprocally connected, particularly in proximity to association cortices.
Repeat steps 3 and 4 until loopy belief propagation converges.	The formation of cross-modal synchronized complexes (at slower beta, alpha, and theta) frequencies may entail loopy message passing across modalities via self-organizing harmonic modes (SOHMs).
Result: Highly reliable data transmission even under highly noisy circumstances.	Result: Highly reliable perceptual inferences from noisy and ambiguous sensory information.

As Bengio (2017) has suggested with his work on the “consciousness prior,” the reduced dimensionality of these (disentangled) features may be well-suited for identifying major axes of meaningful variations in the world, such as those involved in the kinds of causal processes we can manipulate and perceive, and which can also be mapped onto linguistic systems. This later affordance has relevance to Higher-Order Theories of consciousness, including those emphasizing agentic modeling and social communication (Metzinger, 2010; Graziano, 2013; Rudrauf et al., 2017; Brown et al., 2019; Shea and Frith, 2019).

The thalamocortical system enabling dynamic cores of integration and conscious workspaces first evolved hundreds of millions of years before these higher-order processes existed (Edelman, 2004). These richly connected subnetworks enable high-bandwidth message passing—as likely required for realizing turbo codes in biological systems—but are also metabolically expensive, consuming nearly 50% of cortical metabolism in humans (Heuvel et al., 2012). However, part of the way these energetic costs may be justified is by (a) reducing the number of (noisy) neuronal signal transactions required to achieve adequately reliable perceptual inference, (b) enhancing the speed of model selection for the sake of fine-grained control, and (c) allowing for imagination-based planning and causal reasoning (Pearl and Mackenzie, 2018). Rich-club connected subnetworks can even be found in *C. elegans* with their 302 neurons (Towlson et al., 2013). This could be taken to imply that consciousness is nearly a billion years old, but IWMT suggests that this is likely a mistaken inference, as deep hierarchies may be required for generating coherent experience.

### Conscious AI?

IWMT does not suggest that consciousness corresponds to either the output layers of generative models as currently used in machine learning or the processes calculating those outputs. Although architectures with self-attention mechanisms have been implemented with great success (Kovaleva et al., 2019), the outputs of such systems tend to be functionally disconnected from each other, as well as the processes by which they are generated. This is not the case for brains, for which IWMT proposes that joint posteriors and estimates (and samples derived thereof) are calculated via spreading neuronal activity, where message-passing/belief-propagation is promoted (or scheduled) via synchronous dynamics (i.e., SOHMs). As opposed to current generations of generative models, the functioning of these synchronized subnetworks (and the calculations they entail) span multiple levels of hierarchical depth, with bidirectional linkages to generative processes involving models with spatial, temporal, and causal coherence for system and world.

Further, the anatomical and functional directedness of neuronal connections at any point in time contain information that will bias future dynamics, so influencing likelihoods with which meta-stable regimes are subsequently produced. If these networks are altered according to principles of spike-timing dependent plasticity, and if systems develop in the context of embodied agents interacting with their environments, then these state transitions are likely to contain coherent information reflecting causal world structures (Hayek, 1952; Markram et al., 2011; Lakoff, 2014). In these ways and more (e.g. recurrent dynamics persisting across SOHM-formation events), each quale state would functionally connect and constrain future quale states based on past quale states. Further, biological neural networks do not generate feature maps as isolated vectors over stimulus dimensions, but as vectors coupled over multiple levels of hierarchical depth, via neuronal dynamics. Thus, consciousness may be entailed by the functioning of a probabilistic model that generates tensors in neuronal (and representational) phase space, specifying joint posteriors (or estimates derived thereof), where that which is being modeled/estimated is the causes of sensation. If this is the type of mathematical object that corresponds to subjective experience, then substantial progress may have been made toward solving the Hard problem of consciousness.

### Conclusion: Toward (Dis-) Solving the Meta-Problem by Solving the Hard Problem

How could there be “something that it is like” to be a physical system or entailed mathematical object? IWMT suggests that this question may be satisfyingly answered if such a system can calculate—or probabilistically infer—sequences of sensorimotor states. Perhaps intuitively, such a sequential unfolding would have more of a resemblance to the flowing of the stream of consciousness for the kinds of embodied–embedded agents that we are. If models can generate particular combinations of information present within and between sensory modalities, then we may finally begin to have prima facie reasons to believe that such processes could generate subjective experience.

Global workspaces have been analogized as functioning as (non-Cartesian) theaters (Dehaene, 2014) in which information is rendered visible to otherwise isolated modules, with attention acting as a “spotlight” prioritizing some contents over others. Similar metaphors for awareness have been used by Crick and Koch (2003) with their neuronal coalitions model and also by Hobson and Friston (2016) in suggesting that frontal lobe ensembles produce awareness when they “look” at posterior sensory information. While the implication of some sort of little person in the brain, or homunculus, is nearly universally reviled, this dismissal may be a significant part of the Hard problem's intractability. That is, in attempting to do away with homunculi, cognitive science may have lost track of the importance of both embodiment and centralized control structures. If “cognition” is primarily discussed in the abstract, apart from its embodied–embedded character, then it is only natural that explanatory gaps between brain and mind should seem unbridgeable. IWMT, in contrast, suggests that many quasi-Cartesian intuitions may be partially justified. As discussed in Safron (2019a,c), brains may not only infer mental spaces, but they may further populate these spaces with body-centric representations of sensations and actions at various degrees of detail and abstraction. From this view, not only are experiences re-presented to inner experiencers, but these experiencers may take the form of a variety of embodied self-models with degrees of agency. In these ways, IWMT situates embodiment at the core of both consciousness and agency, so vindicating many (but not all) folk psychological intuitions.

With respect to the meta-problem, one could imagine postulating a “Hard problem” of generative models in machine learning, for which an unbridgeable explanatory gap may be perceived between the remarkable ability of these architectures to generate rich and novel stimuli (e.g., an “imagined” face), contrasted with the determinism of their underlying computations. Yet this seemingly intractable problem could then be solved via deeper technical understanding. IWMT proposes that this epistemic situation may be analogous to the one we face with consciousness. Rather than the “Hard problem” being reduced to many “easy problems”—and so being (dis-)solved as we discover we were asking the wrong question—it may be the case of this most challenging and profound problem actually being solved through the discovery of sufficiently powerful bridging principles. IWMT suggests such principles may be finally available by using FEP-AI to integrate leading theories of consciousness.

## Author Contributions

AS conceived and developed this theoretical framework, wrote the entirety of this manuscript, and created all tables and figures therein.

## Conflict of Interest

The author declares that the research was conducted in the absence of any commercial or financial relationships that could be construed as a potential conflict of interest.
