# Supplementary material for: An Integrated World Modeling Theory (IWMT) of Consciousness: Combining Integrated Information and Global Neuronal Workspace Theories With the Free Energy Principle and Active Inference Framework; Toward Solving the Hard Problem and Characterizing Agentic Causation
Source: Front Artif Intell. 2020 Jun 9;3:30. doi: 10.3389/frai.2020.00030 (PMC7861340; doi:10.3389/frai.2020.00030)
Supplement: Supplementary file 1 [file Data_Sheet_1.pdf]

Some potentially testable hypotheses associated with Integrated World Modeling Theory (IWMT):

1. Extensive overlap should be found between neural correlates of consciousness and neural systems implicated in modeling space, time, cause, and selfhood. Disruption of retrosplenial and posterior medial cortices (where these coherence-making properties converge) will be associated with loss of both access and phenomenal consciousness, depending on the extent to which functionality is reduced, indexed by decreased (posterior) alpha coherence. If this were found to not be the case, it would falsify IWMT.
2. Conscious access will depend on subnetworks with high centrality and betweenness. If this were found to not be the case, it would falsify IWMT.
3. Access consciousness should be associated with theta, alpha, and beta oscillations, mediated by long distance signaling from deep (L5) pyramidal neurons and thalamic relays. If this were found to not be the case, it would falsify IWMT. Basic phenomenal consciousness should be associated with alpha and beta frequencies, even if not accompanied by access; although it is difficult to test for phenomenality without access.
4. IWMT suggests that global workspaces constitute complexes with high amounts of integrated information. While the perturbation complexity index (PCI) can potentially be explained without appealing to IIT, it can nonetheless be used as a proxy for integrated information. If GNWT and IIT are compatible in the way suggested here, then PCI should be higher during periods where workspace dynamics are present. This could potentially be tested by timing the TMS pulse to coincide with ignition events during which large scale integration occurs. If this were found to not be the case, it would falsify IWMT.
5. Integrated information appears to be maximized in systems exhibiting the hallmarks of self-organized criticality. If reliable indicators of "edge of chaos" organization (e.g. critical slowing down, fractal dimension) were found to be associated with enhanced learning, this would constitute strong support for the suggested integration of IIT and FEP-AI, wherein phi parameterizes ability to generate model-evidence (or minimize free energy). Further, if critical organization supported ignition events, this would provide support for the suggested integration of IIT and GNWT in terms of modeling workspaces as complexes of integrated information. While IWMT does not depend on these particular hypotheses being accurate, it would constitute strong support for IWMT if they were found to be well-evidenced.
6. Systems with high estimated integrated information should also be more capable of generating model evidence (and minimizing prediction-error). If this were found to not be the case, it would falsify IWMT.

Note: For further testable hypotheses, please see the following preprint:
Safron, A. (2019). Integrated World Modeling Theory (IWMT) Revisited [Preprint]. PsyArXiv. https://doi.org/10.31234/osf.io/kjngh
